# Lysine 63-linked ubiquitination of tau oligomers contributes to the pathogenesis of Alzheimer’s disease

**DOI:** 10.1016/j.jbc.2022.101766

**Published:** 2022-02-22

**Authors:** Nicha Puangmalai, Urmi Sengupta, Nemil Bhatt, Sagar Gaikwad, Mauro Montalbano, Arijit Bhuyan, Stephanie Garcia, Salome McAllen, Minal Sonawane, Cynthia Jerez, Yingxin Zhao, Rakez Kayed

**Affiliations:** 1 Mitchell Center for Neurodegenerative Diseases, University of Texas Medical Branch, Galveston, Texas, USA; 2 Departments of Neurology, Neuroscience and Cell Biology, University of Texas Medical Branch, Galveston, Texas, USA; 3 School of Medicine, University of Texas Medical Branch, Galveston, Texas, USA; 4 School of Dentistry, University of Texas Health Science Center, Houston, Texas, USA; 5 Department of Translational Molecular Pathology, University of Texas MD Anderson Cancer Center, Houston, Texas, USA; 6 Department of Internal Medicine, University of Texas Medical Branch, Galveston, Texas, USA; 7 Institute for Translational Sciences, University of Texas Medical Branch, Galveston, Texas, USA

**Keywords:** Alzheimer’s disease, K63 ubiquitination, tau secretion, tau pathology spreading, proteasome impairment, AD, Alzheimer’s disease, AD TauO, AD brain-derived tau oligomers, ALS, autophagy–lysosome system, Htau, human tau, iHEK-Tau, HEK293 cell line with inducible expression of human 4R tau, LC-MS/MS, liquid chromatography with tandem mass spectrometry, NFTs, neurofibrillary tangles;, PHF, paired helical filament, PLA, proximity ligation assay, TauO, Tau oligomers, UPS, ubiquitin–proteasome system

## Abstract

Ubiquitin-modified tau aggregates are abundantly found in human brains diagnosed with Alzheimer’s disease (AD) and other tauopathies. Soluble tau oligomers (TauO) are the most neurotoxic tau species that propagate pathology and elicit cognitive deficits, but whether ubiquitination contributes to tau formation and spreading is not fully understood. Here, we observed that K63-linked, but not K48-linked, ubiquitinated TauO accumulated at higher levels in AD brains compared with age-matched controls. Using mass spectrometry analyses, we identified 11 ubiquitinated sites on AD brain-derived TauO (AD TauO). We found that K63-linked TauO are associated with enhanced seeding activity and propagation in human tau-expressing primary neuronal and tau biosensor cells. Additionally, exposure of tau-inducible HEK cells to AD TauO with different ubiquitin linkages (wild type, K48, and K63) resulted in enhanced formation and secretion of K63-linked TauO, which was associated with impaired proteasome and lysosome functions. Multipathway analysis also revealed the involvement of K63-linked TauO in cell survival pathways, which are impaired in AD. Collectively, our study highlights the significance of selective TauO ubiquitination, which could influence tau aggregation, accumulation, and subsequent pathological propagation. The insights gained from this study hold great promise for targeted therapeutic intervention in AD and related tauopathies.

Alzheimer’s disease (AD) is the most common cause of dementia worldwide, with its prevalence only expected to double by 2060 barring any advances in the care for the disease (1). The pathological aggregation of the microtubule-associated tau protein and its subsequent deposition in neurofibrillary tangles (NFTs) are defining histopathological features of AD and other neurodegenerative diseases that are collectively known as tauopathies (2, 3, 4). Although tau aggregation is strongly linked to clinical severity and progression of AD, soluble tau oligomers, not the NFTs, are shown to be pathogenetic culprits in AD (5, 6, 7, 8). While the ubiquitin–proteasome system (UPS) and autophagy–lysosome system (ALS) regulate proteostasis and clearance of misfolded protein aggregates, UPS impairment is a key feature of AD (9), resulting in accumulation of higher levels of undegraded ubiquitinated tau proteins (10). Evidence suggests that ubiquitinated tau is a component of pathological lesions in AD brains (11, 12); these tau aggregates can cause proteotoxic stress and exacerbate neuron loss and cognitive decline (10). Soluble tau oligomers have been identified as the more toxic species and most relevant to disease progression (8, 13). Yet, significant knowledge gaps remain in the role of different ubiquitin topologies in pathological tau oligomer processing, assembly, spreading, and degradation. Previous studies have shown tau hyperphosphorylation to be an essential function for pathological tau aggregation (14). However, recent studies have shown that differential ubiquitination of pathological tau can mediate structural diversity and tauopathy strains, which could influence different disease outcomes (15). Therefore, ubiquitination of tau oligomers may represent an important contributor in AD progression. Currently, it is unclear whether different ubiquitin residues contribute to the formation, accumulation, and spreading of tau oligomer pathology.

Ubiquitin is a key regulatory molecule that signals proteins for degradation by the proteasome. Recent research has shown that ubiquitin is involved in other cellular mechanisms aside from proteasomal degradation, such as cell cycle regulation, protein trafficking, autophagy, DNA repair, and nuclear factor-kappa B (NFκB) activation (16). Ubiquitin has seven lysine (K) residues, and differential ubiquitination of these residues determines the cellular outcome. The most predominant ubiquitin linkage is K48, which has been implicated in proteasomal degradation (16). K63-linkage is the second most common, which has been implicated in autophagy (17), and immunoreactivity to K63-linked polyubiquitination is the most common feature of neurodegeneration (18). It is noteworthy to mention that K63-linked ubiquitination is typically not associated with protein degradation (19) while K48 polyubiquitinated proteins are targeted for proteasomal degradation pathway (19). The remaining polyubiquitin linkages are less understood and are not implicated in protein degradation (16).

The primary role of ubiquitination on tau appears to be the clearance mechanism by either the proteasomal or autophagy pathways (20). Petrucelli *et al*. (21) reported that tau ubiquitination by CHIP increases its aggregation and insolubility. Using tandem mass spectrometry, Cripps *et al* demonstrated that insoluble tau in AD paired helical filament (PHF) is ubiquitinated by K48-linkage at microtubule-binding domain, suggesting that tau ubiquitination may play a role in AD progression (21, 22).

Ubiquitination can alter tau function and affect tau self-assembly, aggregation, and accumulation in NFTs (15, 23, 24). The effect of ubiquitination on the function and pathogenesis of tau oligomers is not known. Previously, we have shown that tau oligomers are present in the early stages of neuronal cytopathology in AD (23), and that tau oligomers are secreted extracellularly and can be detected in the CSF (25, 26). This suggests that elevated CSF tau oligomer levels occur early in the course of dementia and may be useful in early diagnosis of AD (23, 27, 28, 29). Further, we also have shown that tau oligomers are only ubiquitinated at earlier stages of aggregation before filamentous NFT formation (23, 25), indicating that ubiquitinated tau converts into more stable and less toxic aggregates. Characterizing the ubiquitination signature of pathological tau oligomers could be helpful for early AD diagnosis.

Using tauopathy cellular, animal models and postmortem brain tissues from patients with AD and age-matched nondemented control cases, this study revealed that ubiquitination influences formation, deposition, and spreading of pathological tau oligomers and contribute to the AD etiology.

## Results

### Hyperubiquitination of tau oligomers associates with the pathological tau aggregation and deposition in the brain of patients with AD

**Figure 1:**
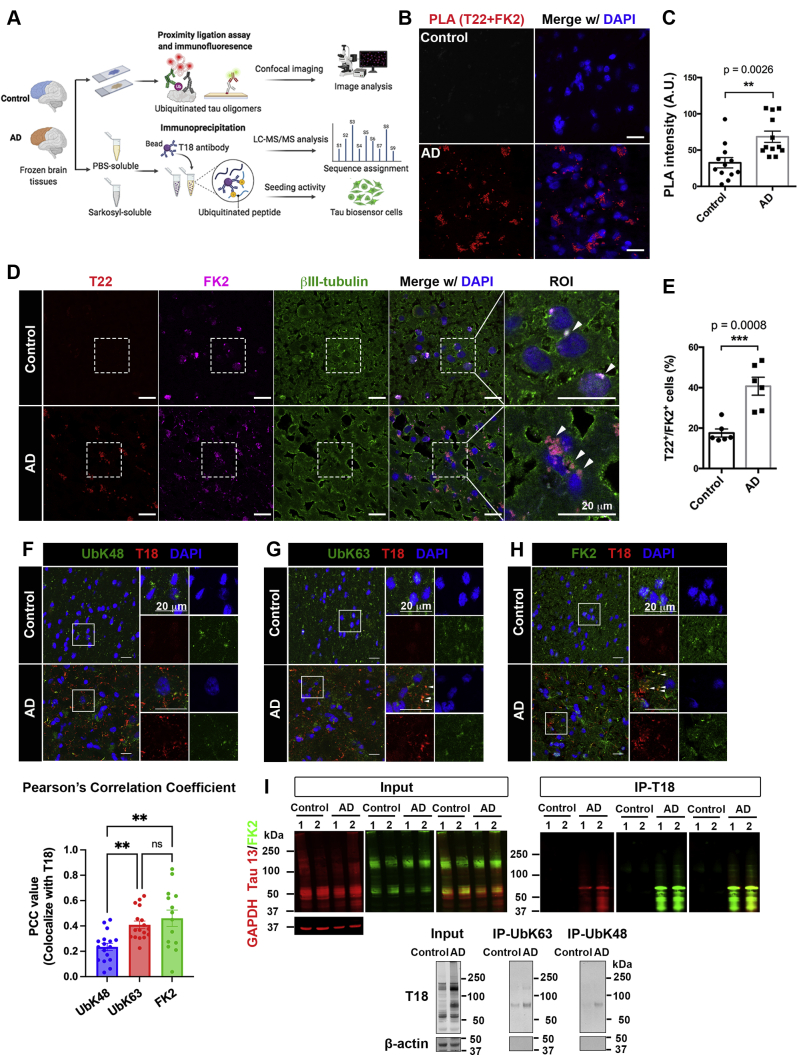
**K63-linkage ubiquitin expression associated with misfolded tau deposition in postmortem AD brains.***A*, schematic of research strategies for oligomeric tau ubiquitination detection in human AD and age-matched control brain tissues. Frozen brain tissue slides from frontal cortices revealed tau oligomer and ubiquitin interaction and colocalization *via* proximity ligation assay (PLA), immunofluorescence (IF), and confocal imaging. Toxic tau aggregates, isolated from PBS-soluble and Sarkosyl (SRK)-soluble fractions followed by immunoprecipitation (IP) with T18 antibody, were analyzed for their ubiquitination profile and seeding activity using LC-MS/MS and Tau biosensor cells, respectively. *B* and *C*, direct interaction of oligomeric tau and ubiquitin in human brain tissues determined by PLA. *B*, complimentary ligation signal (T22 + FK2, *red*) merged with DAPI (*blue*) in control and AD brain samples using anti-T22 and anti-FK2 antibodies. Scale bar = 20 μm. *C*, PLA signal intensity indicated strong interaction of tau oligomers and ubiquitin in AD brain tissues compared to age-matched controls. Statistical analyses were calculated by unpaired and two-tailed Student’s *t* test. Bar graph showed as mean ± SD, N = 3 cases (∗∗*p* < 0.01). *D* and *E*, increase of T22/FK2-positive mature neurons in AD postmortem brain compared to age-matched control. *D*, confocal images showing anti-T22 (*red*), anti-ubiquitin FK2 (*magenta*), and anti-βIII-tubulin (*green*) merged with DAPI (*blue*) in human control and AD brain samples. Scale bar = 20 μm. Region of interest (ROI) marked in white outline and showed at high magnification with 2X digital zoom. Arrow heads indicate colocalization of anti-T22 and anti-ubiquitin FK2 in neurons. N = 3 cases. *E*, bar graph showing percentage of T22/FK2-positive mature neurons in AD postmortem brain tissues vs. age-matched control samples. Statistical analyses were calculated by unpaired and two-tailed Student’s *t* test. Data represented as mean ± SD, N = 3 cases (∗∗∗*p* < 0.001). *F*–*H*, ubiquitin linkage specific on misfolded tau aggregates in AD brain tissues. Confocal images of frontal cortices in control and AD brains presented misfolded tau aggregates (T18, *red*) with (*F*) K48-linked ubiquitin (UbK48, *green*), (*G*) K63-linked ubiquitin (UbK63, *green*), or (*H*) ubiquitinated protein (FK2, *green*). Scale bar = 20 μm. ROIs are outlined in white and showed at 2X digital zoom in separate and combined color channels to *right.* Arrow heads indicate colocalization of T18-positive tau aggregates with K63-linked ubiquitin and generic ubiquitin FK2 in AD brain tissues. PCC analysis measured the colocalization of ubiquitin isoforms with T18-positive tau aggregates in AD brain tissues. Statistical analyses were calculated by One-way ANOVA with Tukey test. Results shown as mean ± SD (N = 3 cases). (∗∗*p* < 0.01). *I*, interaction between tau aggregates and ubiquitin species in controls and AD brain homogenates (N = 2) using immunoprecipitation (IP) with T18 antibody followed by Western blotting with total ubiquitin (FK2) antibody in input and IP fractions. Immunoprecipitated tau with UbK63 or UbK48 antibodies (below panel) confirmed tau and ubiquitin interaction. Tau isolated from IP using UbK63 antibody (MW: > 50 kDa) showed greater intensity of higher aggregates in AD cases compared to controls. GAPDH and β-actin were used as loading controls. AD, Alzheimer’s disease; PLA, proximity ligation assay.

Tau oligomerization is an early and fundamental requisite for tau pathology (23, 30), whether ubiquitin interacts with tau oligomers in AD is not known. To detect the ubiquitination and tau oligomers interaction *in situ* in AD brains, we performed a proximity ligation assay (PLA) using T22 antibody, which specifically recognizes tau oligomers and FK2 antibody, which recognizes ubiquitinated proteins. PLA analysis revealed increased red fluorescence (T22-FK2 positive) signal in AD cortex compared with age-matched controls (Fig. 1, *A*–*C*), indicating that ubiquitinated tau oligomers are abundantly present in AD brains. Moreover, neuronal and glial cells showed tau oligomers colocalization with ubiquitin using a combination of PLA with immunofluorescence staining (Fig. S1, *A*–*C*), suggesting that ubiquitinated tau oligomers are present in both neuronal and nonneuronal cell types. To validate our PLA results, we performed immunofluorescence assay and found an increased number of T22-FK2 positive cells (Fig. 1, *D* and *E*) in AD brain tissues.

### K63-linked-polyubiquitin chain interacts with pathological tau in AD brain tissues and tauopathy mouse models

Next, we examined whether a specific polyubiquitin chain has a preferential interaction with pathological tau deposition in human AD brains. We used T18 antibody that recognizes misfolded tau aggregates (31) with a commercially available Total tau antibody for immunofluorescence and Tau 13 antibody for coimmunoprecipitation (co-IP) assays of AD brain tissues. T18 recognized increased tau aggregates in AD brain tissues compared with controls (Fig. S3*A*). Immunofluorescence and PCC analyses revealed the significantly higher colocalization of misfolded tau aggregates with K63-linked FK2-positive polyubiquitin chains in AD compared with control brains (Fig. 1, *F*–*H*). Surprisingly, there was no different degree of colocalization of T18-positive tau aggregates and K48-linked chains in control and AD brains. Immunolabeling of K63- or K48-linked ubiquitin immunoprecipitated tau aggregates using T18 antibody confirmed that misfolded tau aggregates from AD brain homogenates were ubiquitinated by K63-linked ubiquitin chains, located at above 50 kDa molecular weight (Fig. 1*I*). To explore whether the K63 ubiquitination of tau oligomers was influenced by age, we performed an immunofluorescence assay in hippocampal sections from 2 to 14-month-old Htau mice, which lack endogenous tau (*Mapt*) gene expression and express all six isoforms (including both 3R and 4R forms) of human tau. Increased colocalization of K63-, but not K48-linked, positive tau aggregates was observed in the dentate gyrus in an age-dependent manner (Fig. S1, *D*–*J*). The observations in human AD and Htau mouse brain tissues indicate that pathological tau aggregates are preferentially modified by K63-linked ubiquitination.

**Figure 2:**
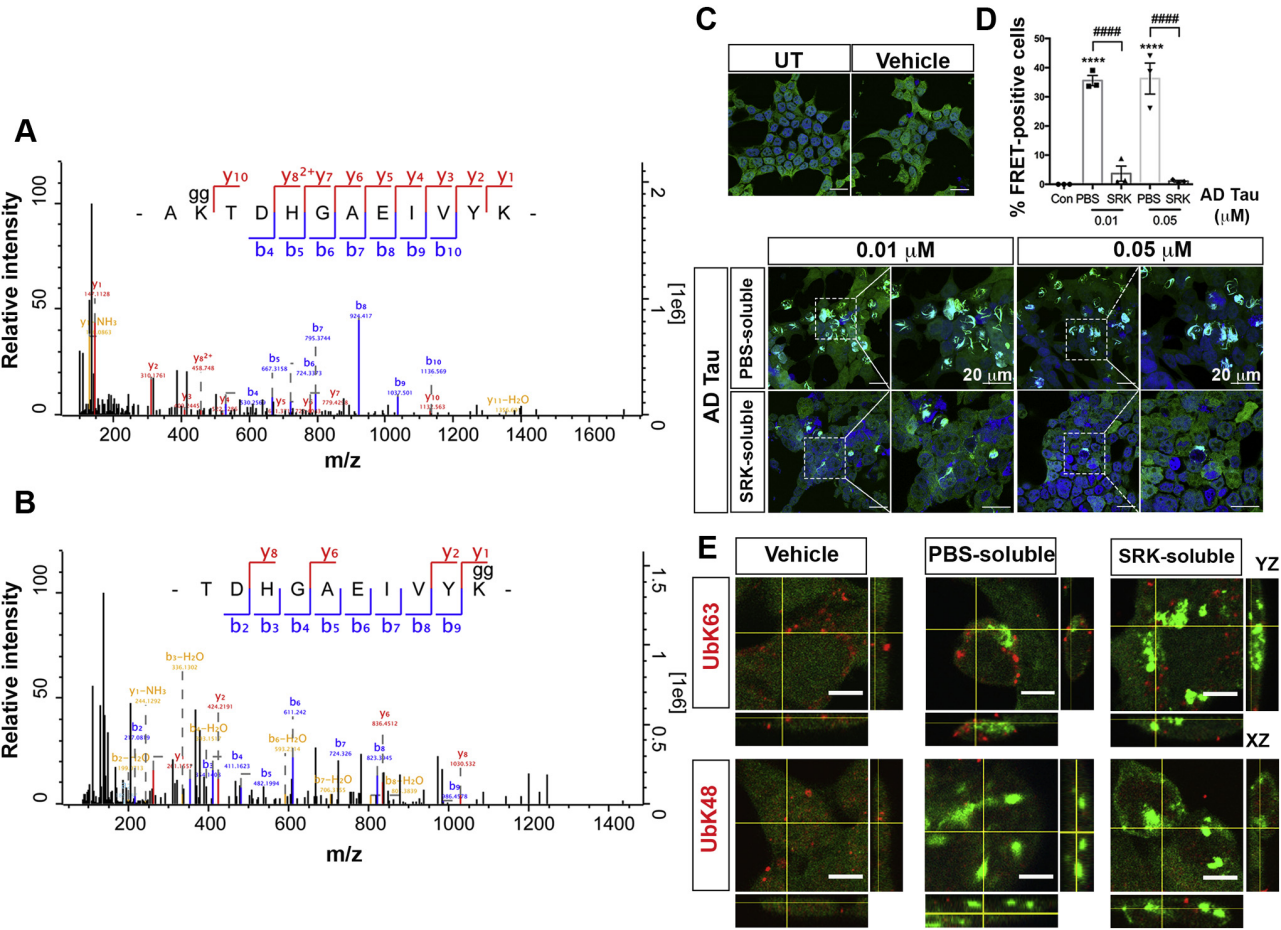
**K63-linked ubiquitination associates with tau aggregate formation in AD brain-derived tau oligomer-exposed tau biosensor cells.***A* and *B*, LC-MS/MS analysis of tau isolated from AD brain tissues detecting ubiquitination sites at the C-terminal region of tau. *A*, annotated MS/MS spectrum of ubiquitinated tau-K385 [AK(gg)TDHGAEIVYK]. Fragment ions (b ions and y ions) were labeled. *B*, annotated MS/MS spectrum of ubiquitinated tau-K395 [TDHGAEIVYK(gg)]. *C*, representative images of PBS- or SRK-soluble tau aggregates from AD brains showed distinct seeding activity between the two fractions after 24 h exposure. ROIs are outlined in white and shown at 2X digital zoom to *right*. Untreated (UT) and vehicle control (Con) (2% v/v lipofectamine) showed in the *top left* panel. Nuclei were counter-stained with DAPI (*blue*). Scale bar = 20 μm. *D*, quantitative analysis of % FRET positive cells were analyzed by counting the FRET positive cells divided by total DAPI positive cells from three random regions (with at least 50 nuclei per image). Statistical analyses were calculated by One-way ANOVA with Tukey test. Bar graphs represent mean ± SD from triplicates (∗∗∗∗*p* < 0.0001 vs. Con., ^###^*p* < 0.001, ^####^*p* < 0.0001 for PBS-soluble compared with SRK-soluble). *E*, tau biosensor cells treated with vehicle-, PBS-soluble or SRK-soluble AD tau aggregates were immunostained with anti-K63-linked ubiquitin (UbK63, *red*) or anti-K48-linked ubiquitin (UbK48, *red*). Scale bar = 5 μm. Representative orthogonal images showed K63-linked ubiquitin surrounded and colocalized with FRET-positive aggregates after PBS-soluble tau treatment. AD, Alzheimer’s disease.

Next, we used LC-MS/MS to identify ubiquitination sites of tau. Tau was immunoprecipitated and digested with trypsin. The ubiquitin was cleaved from tau, and two glycine residues remained on the lysine residues of tau. We identified 11 ubiquitination sites and two acetylation sites of tau (Table S1). Consistent with previously identified ubiquitinated sites in human AD brain (32), we also detected ubiquitin sites at K259 and K267 at R1 and R4 repeat sequences of tau isolated from human AD brain tissues. Additionally, ubiquitination of K385 and K395 at C-terminal region of tau was detected, and the MS/MS spectra of ubiquitinated tau-K385 and tau-395 are shown in Figure 2, *A* and *B*, respectively. The annotated MS/MS spectra of other ubiquitinated or acetylated tau peptides are shown in Fig. S7. Interestingly, these tau ubiquitin sites have been previously mapped in paired helical filament-enriched tau fractions from AD brains and mouse tau from transgenic mice overexpressing APP (32, 33, 34, 35). These findings suggest that tau ubiquitin sites are exclusively preserved on microtubule-binding domains and the C-terminal region, independent of tau aggregation.

### K63-linked ubiquitination associates with enhanced seeding activity of AD-brain-derived tau oligomers

We next examined whether ubiquitination may modulate tau seeding activity in AD pathology. To do so, co-IP of tau aggregates using T18 antibody was performed from PBS-insoluble fractions of AD brain homogenates in addition to PBS-soluble fractions containing oligomeric tau and higher-order tau aggregates, respectively. Tau biosensor cells were used to detect the seeding activity from isolated tau as previously published (36, 37, 38). Drastically higher number of FRET-positive cells, an indicator of the tau seeding activity, was detected when cells were exposed to purified PBS-soluble tau aggregates compared with PBS-insoluble tau and untreated cells (Fig. 2, *C* and *D*). FRET-positive aggregates in cells treated with PBS-soluble tau had different morphology from the PBS-insoluble tau treated group. We further asked whether the seeded aggregates in tau biosensor cells were associated with a polyubiquitin chain. We performed immunofluorescence staining with ubiquitin linkage specific K48 and K63 antibodies in treated tau biosensor cells. Orthogonal views generated by confocal imaging demonstrated that K63-linked chains were surrounded and partially colocalized with seeded tau aggregates in the PBS-soluble and the insoluble tau-treated group, while K48-linked chains did not show such colocalization with the seeded aggregates (Fig. 2*E*). These findings further suggest that K63-linked polyubiquitin chains are preferentially present in tau aggregate formation after exposure with soluble tau oligomers from AD brains. In contrast, the seeding activity of higher-order tau aggregates might be mediated by other polyubiquitin linkages, which have not been investigated here.

### K63-ubiquitinated AD-TauO enhances tau release and propagation of tau pathology *in vitro*

**Figure 3:**
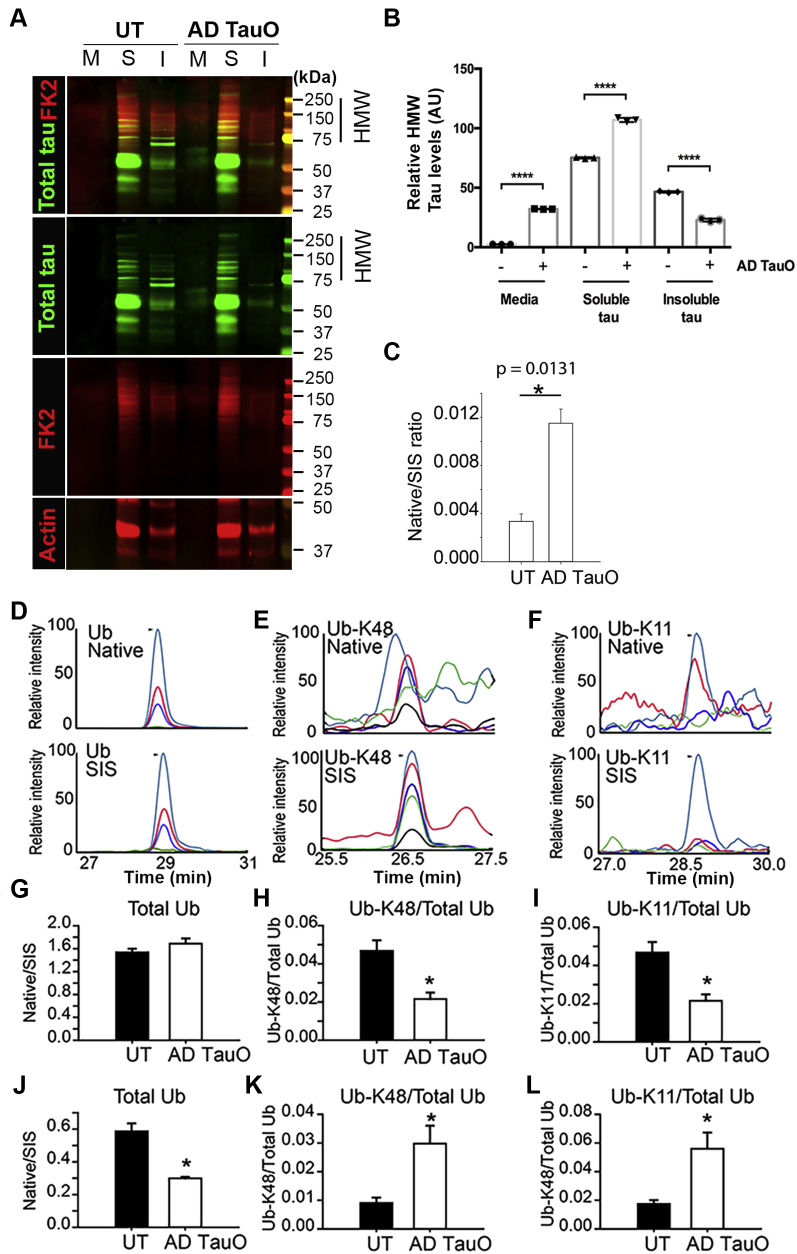
**Effect of ubiquitination on tau release and aggregateformation.***A*–*C*, Western bot analysis of tau from cultured media (M), PBS-soluble (S), and PBS-insoluble (I) fractions detected by anti-total tau (*green*) and anti-ubiquitin (FK2; *red*) antibodies. β-actin (*red*) was used as loading controls for S and I. Samples were loaded equally at 10 μg. *B*, quantification of tau at high molecular weight range (HMW: 75–250 kDa) showed increased levels of tau in culture media (M) PBS-soluble (S) fractions after treatment with AD TauO *versus.* untreated (UT). Bar graphs represent mean ± SD from triplicates. Statistical analyses were calculated by unpaired and two-tailed Student’s *t* test (∗∗∗∗ *p* < 0.0001). *C*, SRM-MS quantification of total ubiquitin on tau from culture media was significantly higher in AD TauO treated compared with UT. Bar graphs represent mean ± SD. Statistical analyses were calculated by unpaired and two-tailed Student’s *t* test (∗ *p* < 0.05). *D*–*L*, SRM analysis of ubiquitin-linked isoforms in tau fractions from AD TauO-treated iHEK-Tau cells. *D*, SRM chromatogram of Ub native peptide from ubiquitin (*upper panel*) and its stable isotope labeled standard (SIS, *lower panel*). Each plot represents the extract ion chromatogram of one SRM transition (y9 teal, y10, *red*, y11, *blue*, and y7 *green*). *E*, SRM chromatogram of native peptide of Ub-K48-linkage from poly-Ub (*upper panel*) and its stable isotope labeled standard (*lower panel*). Each plot represents the extract ion chromatogram of one SRM transition) (y8 *teal*, y6, *red*, y9, *blue*, y5 *green*, and y7 *black*). *F*, SRM chromatogram of native peptide of Ub-K11-linkage from poly-Ub (*upper panel*) and its stable isotope labeled standard (*lower panel*). Each plot represents the extract ion chromatogram of one SRM transition (y9 *teal*, y10, *red*, y11, *blue*, and y8 *green*). *G*, SRM-MS analysis of total ubiquitin on soluble tau. *H*, SRM-MS analysis of Ub-K48-linkage on soluble tau. *I*, SRM-MS analysis of Ub-K11 on soluble tau. *J*, SRM-MS analysis of total ubiquitin on insoluble tau. *K*, SRM-MS analysis of Ub-K48-linkage on insoluble tau. *L*, SRM-MS analysis of Ub-K11 on insoluble tau. The level of total ubiquitin and Ub-linkages were normalized with the total tau, mean ± SD (∗*p* < 0.05). Statistical analyses were calculated by unpaired and two-tailed Student’s *t* test. AD TauO, AD brain-derived tau oligomers.

In AD pathology, soluble tau and insoluble tau from PHFs act as substrates for E3 ubiquitin ligase-mediated ubiquitination and proteasome degradation *via* K11, K48, and K6-linked polyubiquitin chains (12, 17, 22, 39). To examine whether ubiquitination may contribute to the increased release and formation of tau aggregates, we treated iHEK-tau cells with AD TauO and measured tau and ubiquitin levels in cultured media (M), PBS-soluble (S), and PBS-insoluble (I) fractions of cell lysates. Western blot analysis using anti-Total tau and FK2 antibodies indicated that AD TauO not only induced tau release into cultured media, but also increased tau levels in soluble fractions compared with insoluble fractions (Fig. 3, *A* and *B*). We performed SRM-MS analysis in concentrated cultured media to detect the ubiquitination of released tau. A higher quantity of ubiquitinated tau in AD TauO-treated group was observed compared with untreated (UT) (Fig. 3*C*). Surprisingly, we did not detect any interubiquitin linkages chain on tau, suggesting that monoubiquitination might be involved with tau release in AD pathology.

We further investigated polyubiquitin chains in the S and I cell fractions using SRM-MS analysis. The signature peptides and selected reaction monitoring (SRM) transitions of tau, ubiquitin, and ubiquitin linkages were listed in Table S2. Relative intensity profiles of native and stable isotope labeled standard (SIS) of total ubiquitin (Fig. 3*D*), K48-linked (Fig. 3*E*), and K11-linked chains (Fig. 3*F*) were generated. MS analysis of tau in S (Fig. 3, *G*–*I*) and I (Fig. 3, *J*–*L*) cell fractions showed marked decrease of K48 and K11-linked chains, the major ubiquitin isoforms linked to the proteasome degradation pathway (40), found in S fractions. On the contrary, these isoforms were significantly increased in I fractions, suggesting that the binding of K48- and K11-linkages on insoluble tau aggregates is a dominant trait after treatment with AD TauO.

**Figure 4:**
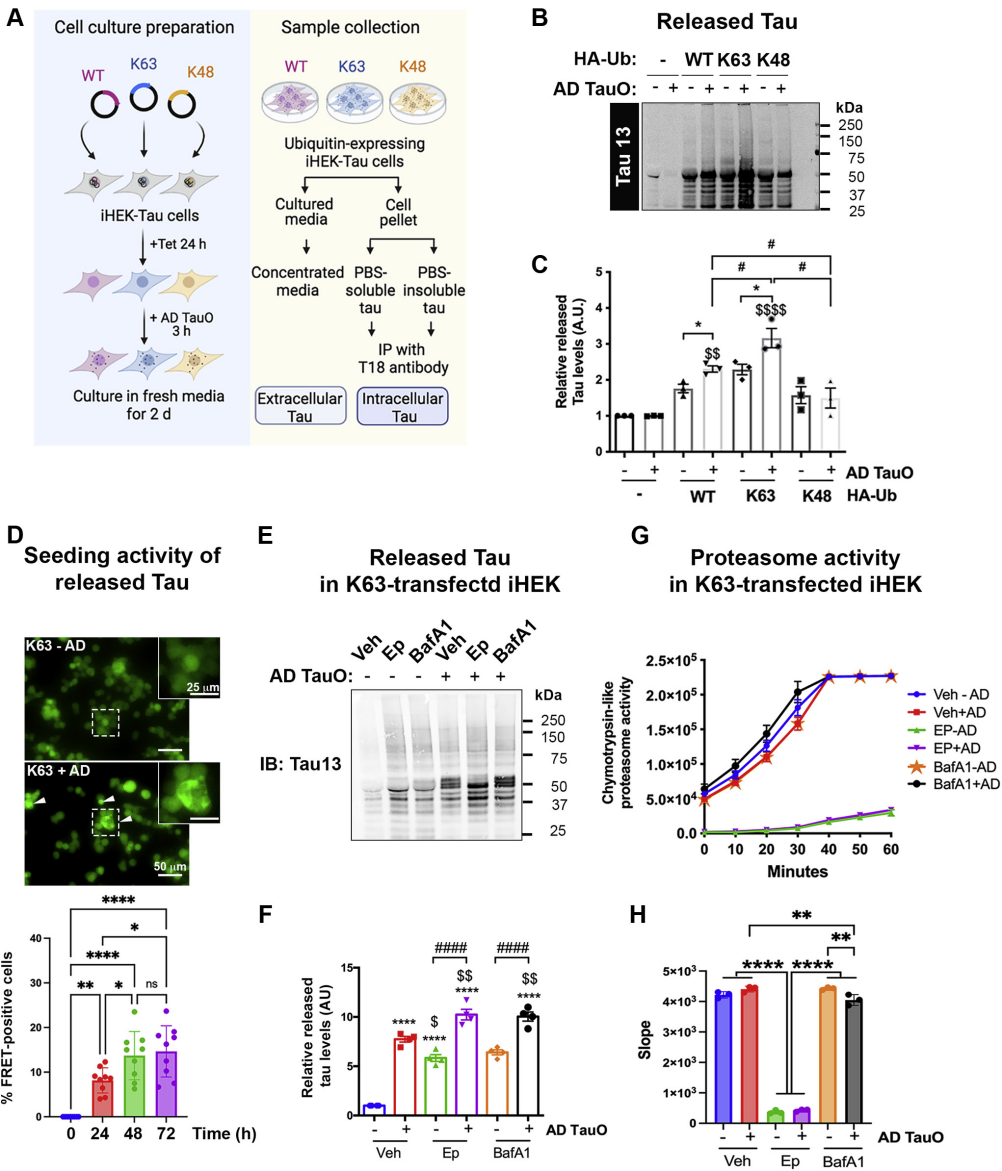
**K63-linked ubiquitin associated with AD TauO-induced tau release in iHEK-Tau cells.***A*, diagram presenting experimental designs of ubiquitin isoform-mediated tau release. iHEK-Tau cells were transfected with DNA plasmid expressing HA-tagged WT, K63-linked (K63), or K48-linked (K48) ubiquitin followed by tau induction with tetracycline (Tet) for 24 h. Cells were treated with 0.1 μM AD TauO for 3 h before culturing in fresh media for 2 days. Extracellular tau was collected from concentrated cultured media. Intracellular tau was isolated from PBS-soluble and insoluble fraction and IP with T18 antibody. *B* and *C*, K63-linked ubiquitin associated with tau release. *B*, cultured media from experimental design (*A*) were detected for total tau (anti-Tau 13) by Western blotting. *C*, quantification of released tau (MW ≥ 50 kDa) was analyzed from three independent experiments. Bar graph shows mean ± SD. Statistical analyses were calculated by One-way ANOVA with Tukey test. (^$$^*p* < 0.01, ^$$$$^*p* < 0.0001 compared to untreated cells; ∗*p* < 0.05 compared to untreated HA-Ub-transfected cells, ^#^*p* < 0.05 compared to HA-Ub-transfected cells with AD TauO treatment). *D*, released tau from K63-linked polyubiquitin chain overexpressing cells contain seeding property. Representative live imaging and quantification of %FRET-positive cells over 72 h of tau biosensor cells treated with concentrated media from K63-linkage overexpressing iHEK-Tau cells with or without AD TauO exposure as mentioned in Figure 4*A*. Scale bar = 50 μm. ROIs in rectangles showed at 2X digital zoom on top right corners. Arrow heads showed FRET-positive cells. Bar graph was shown as mean ± SD from three independent experiments. Statistical analyses were calculated by One-way ANOVA with Tukey test (∗ *p* < 0.05, ∗∗ *p* < 0.01, ∗∗∗∗*p* < 0.0001). *E*–*H*, inhibition of protein degradation pathways exacerbated AD TauO-induced tau release and reduced proteasome activity. K63-linkage-transfected iHEK-tau cells were prepared and treated as shown in experimental design (*A*). At 24 h prior to sample collection, cells were incubated with 0.1 μM proteasome inhibitor (epoxomicin (Ep)), or lysosome/autophagy inhibitor (Bafilomicin A1 (BafA1)). 0.01% DMSO was used as vehicle (Veh). *E* and *F*, immunoblot analysis of total tau (anti-Tau 13) from concentrated media was measured and showed as mean ± SD (∗∗∗∗*p* < 0.0001 compared with Veh; ^$^*p* < 0.05, ^$$^*p* < 0.001 compared with AD TauO-treated cells; ^####^*p* < 0.0001 compared with inhibitor alone). *G*, cell lysate was measured for chymotrypsin-like proteasome activity every 10 min for 1 h at 37 °C and plotted as a response curve. *H*, slopes of proteasome activity were shown as bar graph as mean ± SD from triplicate. (∗∗ *p* < 0.01, ∗∗∗∗*p* < 0.0001). All statistical analyses were calculated by One-way ANOVA with Tukey test. AD TauO, AD brain-derived tau oligomers.

To identify whether any specific ubiquitin linkage is involved in tau release, we performed the overexpression of wild-type (WT), K63, or K48 ubiquitin chains (Fig. 4*A*) in iHEK-Tau cells. AD TauO treatment was used for triggering extracellular tau release. Western blot analysis for tau and ubiquitin levels in concentrated cultured media revealed that AD TauO induced tau release in WT ubiquitin overexpressing cells. Interestingly, K63 ubiquitin overexpressing cells had greater amount of tau release compared with WT, K48 ubiquitin, and untreated cells (Fig. 4, *B* and *C*). However, this response was less detectable in K48 ubiquitin-overexpressing cells (Fig. 4, *B* and *C*). Silver staining of cultured media verified that AD TauO induced an increased amount of protein release from cells with K63 ubiquitin overexpression (Fig. S2*A*). Surprisingly, released tau from AD TauO-treated K63-ubiquitin overexpressing cells showed seeding activity over 72 h in tau biosensor cells (Fig. 4*D*), whereas released tau from WT and K48-linkage overexpressing cells did not have similar seeding property (Fig. S2*D*). These results suggest that K63-linked polyubiquitin chains have an important role in pathological tau propagation in AD pathology.

Toxic tau oligomers found in many neurodegenerative diseases potently inhibit 20S and 26S proteasome gate opening, thus drastically impairing its function (41). Therefore, we further investigated whether K63-ubiquitin overexpression in iHEK-Tau cells treated with AD TauO disrupted mechanisms of protein degradation, including the UPS or the ALS pathways. Hence, we used pharmacological inhibitors such as epoxomicin (Ep) and bafilomycinA1 (BafA1) to inhibit the functions of the proteasome or lysosome/autophagy, respectively, prior to treatment of AD TauO and cell collection. Western blot analysis of concentrated cultured media using Tau 13 showed that inhibition of either proteasome or lysosome/autophagy function per se was able to induce tau release. However, application of AD TauO significantly increased tau release at similar levels from EP- or BafA1-exposed cells (Fig. 4, *E* and *F*). We also observed similar trends of tau release in WT- and K48-ubiquitin overexpressing cells, but to a lesser extent than that of K63-ubiquitin expressing cells (Fig. S2, *B* and *C*). To confirm these results, proteasome activity of K63-linkage overexpressing-iHEK-Tau cells was evaluated by measuring chymotrypsin-like proteasome activity from the cell lysates. Figure 4, *G* and *H* (chymotrypsin-like proteasome activity curves together with a micrograph of slopes) demonstrate that application of AD TauO marginally induced proteasome activity in the vehicle-treated group, which was inhibited by Ep treatment. Interestingly, cells treated for lysosome inhibition had significantly diminished proteasome activity after AD TauO exposure compared to untreated or vehicle-treated groups. These findings suggest that AD TauO mediates tau release and soluble tau aggregate formation *via* activation of K63-linkage and mitigation of K48- and K11-linked chain functions. Dysregulation of proteasome and lysosome functions by AD TauO may be the result of overexpression of K63 ubiquitin chains.

### Differential distribution of pathological tau aggregates linked to ubiquitin-linkages expression in iHEK-Tau cells

**Figure 5:**
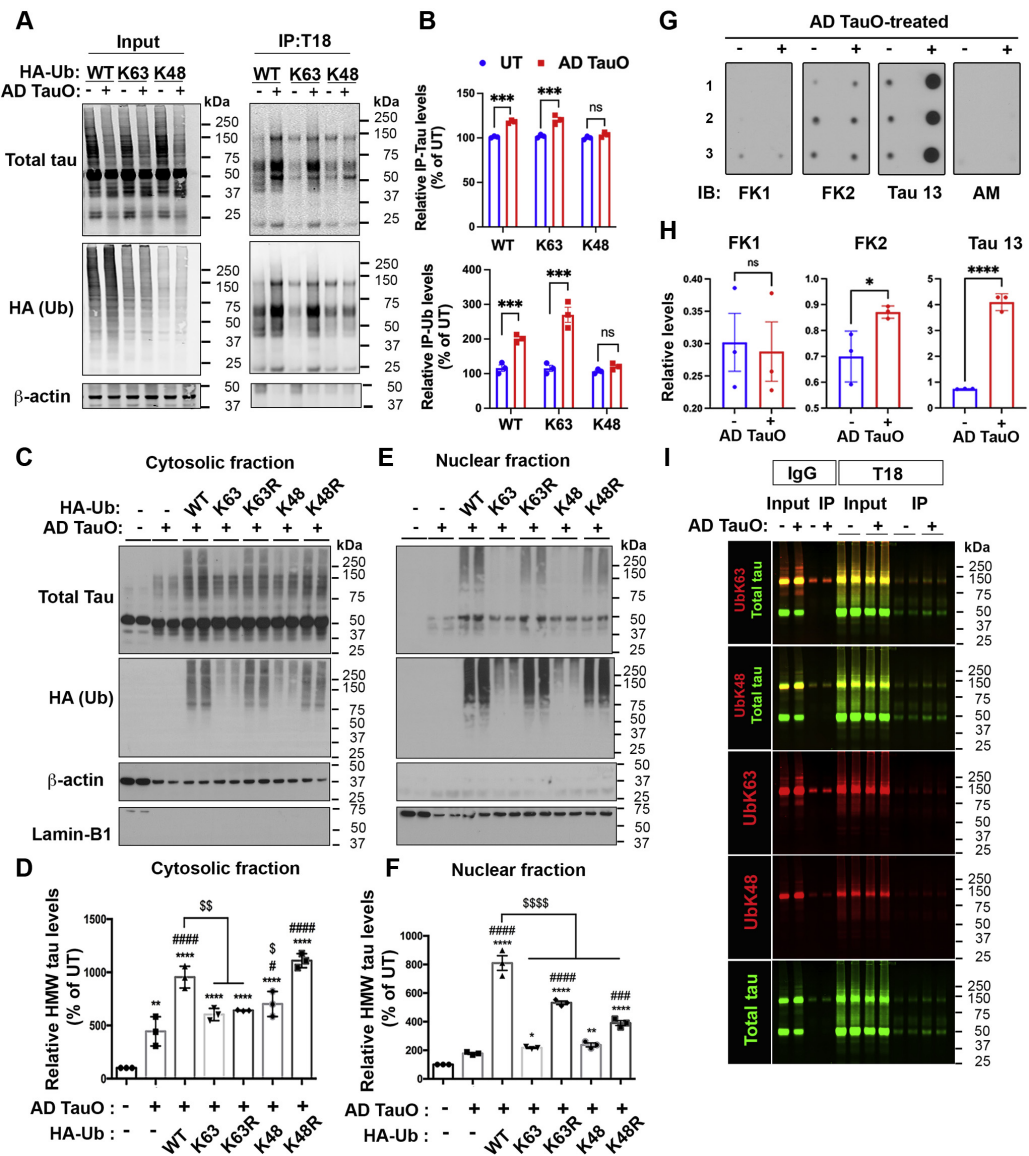
**K63-linked ubiquitin interacts with the formation of misfolded tau aggregates and tau release.***A* and *B*, K63-linked ubiquitin interacted with soluble tau after AD TauO exposure. iHEK-Tau cells were transfected and treated as mentioned in Figure 4*A*. *A*, IP of PBS-soluble tau with anti-T18 antibody followed by WB was measured for total tau and ubiquitin isoform (anti-HA). β-actin served as loading control. *B*, quantification of IP-Tau (*upper*) and Ub (*lower*) levels shown as mean ± SD from three independent experiments using the One-way ANOVA with the Tukey test. (∗∗∗∗*p* < 0.0001 *versus.* UT). *C*–*F*, K63- and K48-linked ubiquitin isoforms related to AD TauO-induced tau aggregates in cytosolic distribution. *C* and *E*, iHEK-Tau cells were transfected with WT-, K63-, K48-linked Ub plasmids before Tet induction with (+) or without (−) AD TauO treatment (0.1 μM, 3 h before culturing in fresh media for 24 h). Ub plasmid-expressing mutant K63 (K63R), and K48 (K48R) were used as negative controls for K63 and K48, respectively. Cell fractionation isolated cytosolic and nuclear proteins and were detected for total tau (anti-total tau), ubiquitin isoforms (anti-HA), and loading controls of cytosol (β-actin) and nuclei (Lamin-B1) fractions. *D* and *F*, quantification of tau expression (MW: ≥ 50 kDa) in cytosolic (*D*) and nuclear (*F*) fractions shown as mean ± SD from three independent experiments using the One-way ANOVA with Tukey test. (∗*p* < 0.05, ∗∗*p* < 0.01, ∗∗∗∗*p* < 0.0001 compared with UT; ^###^*p* < 0.001, ^####^*p* < 0.0001 compared with AD TauO-treated; ^$^*p* < 0.05, ^$$^*p* < 0.001, ^$$$$^*p* < 0.0001 compared with AD TauO-treated WT Ub-transfected cells). *G*–*H*, dot blot analysis and quantification of cultured media from AD TauO-treated MAPT primary neurons. Dot blots probed with anti-polyubiquitinated protein FK1 antibody, anti-mono- and polyubiquitinated protein FK2 antibody, and anti-Tau 13 antibody showed increased tau- and FK2-positive fractions in AD TauO-treated group respective to UT (-). Anti-mouse antibody (AM) was used as a negative control. Statistical analyses were calculated by unpaired and two-tailed Student’s *t* test (∗*p* < 0.05, ∗∗∗∗*p* < 0.0001 compared to UT (-)). *I*, AD TauO induced intracellular misfolded tau ubiquitination by K63-linked ubiquitin in MAPT primary neurons. Co-IP of PBS-soluble tau fraction with antibody against pathological tau T18 antibody followed by WB showed direct interaction of misfolded tau aggregates with K63-linked ubiquitin. AD TauO, AD brain-derived tau oligomers; iHEK-Tau, HEK293 cell line with inducible expression of human 4R tau.

To uncover the effect of ubiquitin on the distribution of tau aggregates induced by AD TauO, we first investigated the influence of specific ubiquitin linkage on intracellular misfolded tau aggregation. Treatment of AD TauO in iHEK-Tau cells with ubiquitin overexpression, including WT, K63, K48, was performed for 24 h prior to IP with anti-T18 antibody from total cell lysate. Validation of HA-ubiquitin isoform expression was shown in Fig. S5. Immunoblot with anti-Total tau and anti-HA antibody for ubiquitin was carried out and analyzed (Fig. 5*A*). Immunoprecipitation revealed a correlative increased tau ubiquitination by WT and K63-linkage after AD TauO exposure (Fig. 5, *A* and *B*), whereas K48-linkage tended to conjugate with misfolded tau but not significantly.

Next, we isolated cytosolic and nuclear fractions from AD TauO treated iHEK-Tau cells followed by Western blot analysis and quantification for total tau and ubiquitinated tau. K63R and K48R ubiquitin mutants were used as negative controls for K63 and K48 ubiquitin, respectively. β-actin and Lamin-B1 were used to validate the protein purity from cytosolic and nuclear fractions, respectively. Results showed that AD TauO induced tau aggregate formation (molecular mass at ≥ 50 kDa) that was present mostly in the cytosol (Figs. 5, *C* and *D*, S6) and modestly enhanced monomeric tau expression in the nuclei (Fig. 5, *E* and *F*). High-molecular-weight tau (HMW ≥75 kDa) was drastically expressed in both cytosolic and nuclear fractions in WT-ubiquitin overexpressing iHEK-Tau cells compared with only AD TauO-treated iHEK-Tau cells (Fig. 5, *C* and *D*). In contrast, HMW tau was remarkably lower in both the fractions of K63 and K48 ubiquitin overexpressing cells. These findings suggest that the interaction of K63 and K48-linked ubiquitin chains with tau aggregates occurs exclusively in the cytosol, while other ubiquitin isoforms may be responsible for the formation of tau aggregates and their translocation to the nuclei, which we have not investigated in this present study.

### K63-linked ubiquitination interplays with the neuronal tau release and pathological tau formation in human tau-expressing primary neurons

To verify our findings in the iHEK-Tau cell line in neuronal cells, primary cortical neurons isolated from Htau-expressing embryos were used to observe tau release after treatment with AD TauO using dot blot analysis. Surprisingly, concentrated cultured media from treated neurons showed increased positive signals for mono- and polyubiquitination using FK2 antibody compared with UT. However, FK1-positive signal for polyubiquitination was scarce (Fig. 5, *G* and *H*). These results suggest that ubiquitin chain of distinct length, particularly, monoubiquitin, perhaps is associated with released neuronal tau. Similarly, MS analysis of released tau from AD TauO-treated iHEK-Tau cells also revealed only ubiquitin with no branches at lysine residues, indicating that tau monoubiquitination plays a role in extracellular tau secretion. Likewise, a significantly higher level of total tau was detected using anti-Tau 13 in AD TauO-treated cells compared with UT (Fig. 5, *G* and *H*), suggesting that AD TauO maintains the pathological seeding property by causing tau release and propagation in neuronal and nonneuronal cells.

Next, IP from primary neuron lysates with T18 followed by Western blot analysis revealed that neuronal tau aggregates triggered by AD TauO strongly interacted with K63-linked ubiquitin (yellow) (Fig. 5*I*), and HMW tau levels were notably enhanced in these cells relative to UT. The interaction of pathological tau aggregates with ubiquitin linkages is evaluated by immunofluorescence analysis of AD TauO-treated primary neurons in Fig. S3, *B* and *C*, which showcase the colocalization of anti-TTCM1 antibody, an in-house monoclonal antibody for toxic tau aggregates (Fig. S3, *D*–*G*), with K63-linked ubiquitin (arrow heads), but not with K48-linked ubiquitin, after AD TauO exposure. Together, these observations suggest that K63-linked ubiquitination plays an important role in tau release and pathological tau aggregate formation in neuronal and nonneuronal cells.

### K63- linkages activate NFκB and JAK/STAT pathways in AD TauO-induced pathology *in vitro*

**Figure 6:**
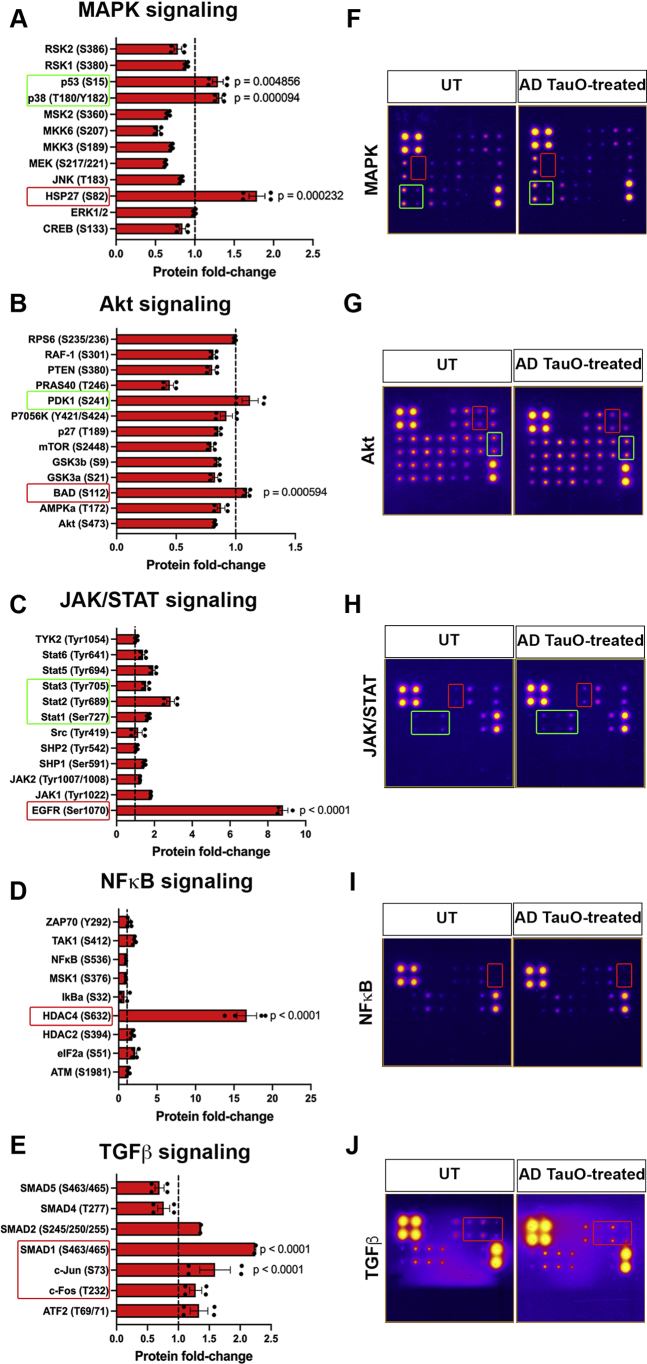
**K63-linkages activate NFκB and JAK/STAT pathways in AD TauO-induced pathology *in vitro*.***A*–*E*, MAPK phosphorylation profiles in AD TauO-treated K63Ub-iHEK-Tau cells. Cell signaling profile in lysate of iHEK-Tau cells with K63Ub overexpression and control cells was measured with RayBio Human phosphorylation profile array. Bar graphs present protein fold-change of signaling activity involved in (*A*) MAPK, (*B*) Akt, (*C*) JAK/STAT, (*D*) NFκB, and (*E*) TGFβ pathways. Statistical analysis was calculated by One-way ANOVA with Tukey test. (∗*p* < 0.05 compared to UT). *F*–*J*, representative chemiluminescent arrays showed in fire lookup tables of (*F*) MAPK, (*G*) Akt, (*H*) JAK/STAT, (*I*) NFκB, and (*J*) TGFβ for UT and AD TauO-treated K63-linkage overexpressing-iHEK-Tau cells. The intensities of array dots were quantified in quadruplet per array and were normalized against the positive controls on the blots. AD TauO, AD brain-derived tau oligomers; iHEK-Tau, HEK293 cell line with inducible expression of human 4R tau.

To gain more insight into the underlying mechanisms of K63 ubiquitination in AD pathology, AD TauO-treated iHEK-Tau cells with K63-linkage overexpression were analyzed for the mechanisms involved in cell survival, inflammatory, and stress response including the mitogen-activated protein kinase (MAPK), protein kinase B (Akt), Janus kinase-signal transducer and activator of transcription (JAK/STAT), nuclear factor-kappaB (NFκB), and transforming growth factor beta (TGFβ) (Figs. 6 and S4, *A*–*E*). Pathway profiles were generated from cells treated with AD TauO with respect to untreated. Quantification of relative protein fold change to untreated cells demonstrated that HSP27 (S82), p38 (T180/Y182), and p53 (S15) in the MAPK cascade were significantly increased, whereas phosphorylation of JNK, MEK, and MKK isoforms and the ribosomal S6 kinase (RSK) family was downregulated (Figs. 6, *A* and *F*, S4*A*). Application of AD TauO drastically diminished the activation of PI3K/Akt/mTOR pathways such as Akt, GSK3a and b, mTOR, p27, PRAS40, PTEN, and RAF-1, while the Bcl-associated agonist of cell death (BAD) protein, a proapoptotic member of the Bcl-2 gene family, which is involved in initialing apoptosis (42), was markedly enhanced (Figs. 6, *B* and *G*, S4*B*). Interestingly, we found that the activation of epidermal growth factor receptor (EGFR) was highly increased as well as the JAK isoforms, SHP1, and Stat protein family (Figs. 6, *C* and *H*, S4*C*). Compared with untreated cells in the NFκB and TGFβ panels, the expression of histone deacetylase 4 (HDAC4), a global regulator for the transcription of genes involved in synaptic plasticity, neuronal survival, and neurodevelopment (43), as well as the function of SMAD1 and SMAD2, showed dramatic increase by AD TauO (Figs. 6, *D*, *E*, *I* and *J* and S4, *D* and *E*). These observations on K63-linkage-expressing cells suggest that AD TauO activates multiple kinases that have been reported in AD pathological conditions (44, 45), referring that the pathology may be due to the overexpression of K63-linked ubiquitin function on tau oligomer.

## Discussion

**Figure 7:**
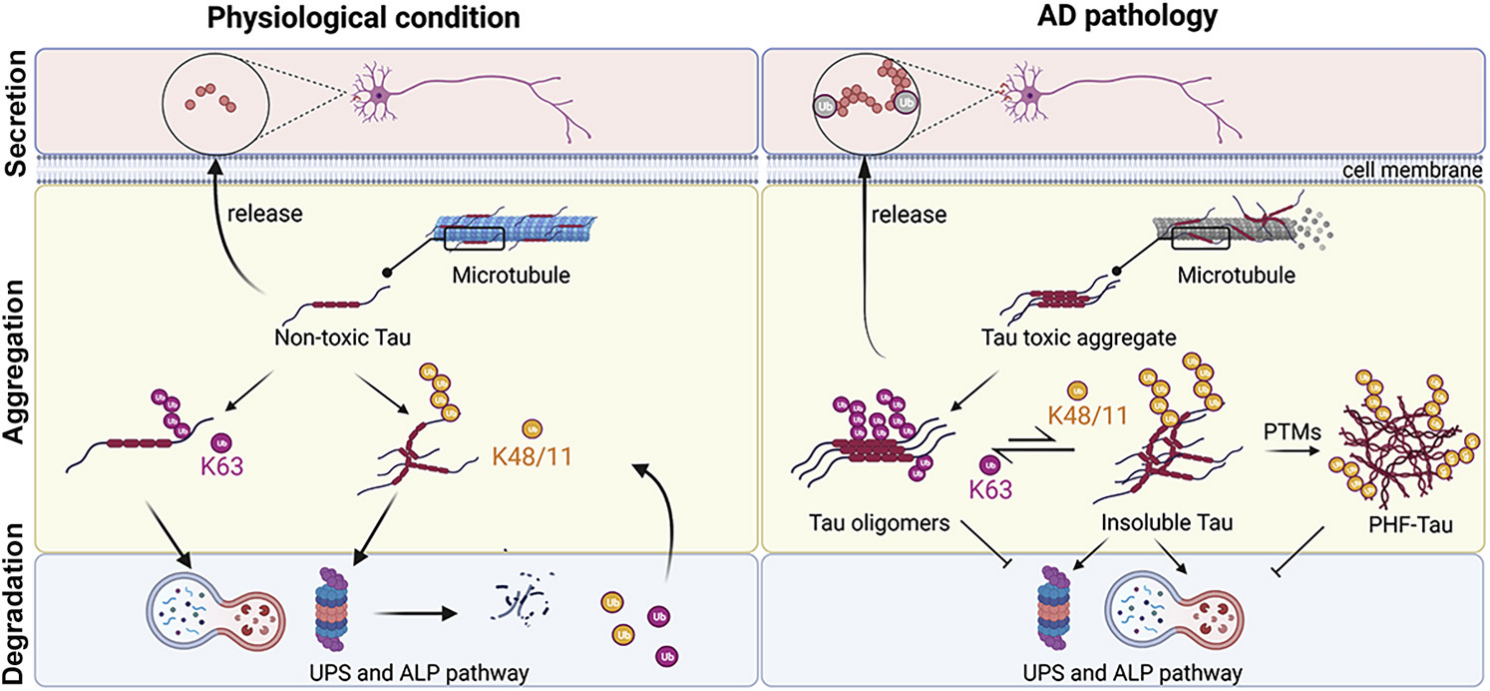
**The role of ubiquitination in tau oligomer pathogenesis in AD.** In physiological condition, tau protein, an axonal microtubule-associated protein, accumulates both intracellularly and extracellularly. Ubiquitin signaling regulates the fate of tau protein by linking lysine (K) 48-, K11- or K63-linked ubiquitin chains for degradation through the ubiquitin-proteasome system (UPS) or the autophagy-lysosomal pathway (ALP). In Alzheimer’s disease (AD) pathology and other tauopathies, abnormal accumulation of misfolded tau aggregates is observed. Tau protein undergoes several posttranslational modifications (PTMs), including K48-, K11-, and K6-linked ubiquitination, and hyperphosphorylation, etc., initiating the formation of degradation-resistant paired helical filaments (PHFs), a key pathological hallmark of AD. We propose that K63-linked ubiquitin, but not K48-linked, selectively binds to soluble tau oligomers to enhance its secretion and spread the pathology as well as attenuate the UPS and ALP pathways. On the other hand, K48-, K11-linked ubiquitin bind to insoluble tau aggregates and subsequently translocate to UPS for degradation.

We have identified an association of K63-linked ubiquitin with pathological tau in postmortem AD brain tissues, particularly in the soluble fraction containing the most toxic oligomeric tau form (Fig. 7) (46, 47, 48), which has not been studied before. To identify the signature peptides of ubiquitination on tau oligomer, a combination of immunoprecipitation with a highly sensitive and specific mass spectrometry method using SRM demonstrated that tau was ubiquitinated at microtubule-binding regions (MTBR) and the C-terminal domains including K254, K311, K353, K385, and K395 residues. Our observation is in accordance with previous studies (12, 22, 32). These tau sites have been reported in the PHFs from AD brains (14), which are specifically monoubiquitinated at K254, K257, K311, and K317 (12) and polyubiquitinated at K254, K311, K353 residues (22). The C-terminal domain of tau ubiquitinated at K385 and K395 has also been reported in Nonidet P-40-soluble tau from AD brains (32). Several mass spectrometry studies suggest that MTBR of tau is enriched in aggregates in AD brain (26, 49, 50). A series of cryogenic electron microscopy with mass spectrometry studies demonstrates a subsegment of the MTBR and the C-terminal domain, comprising the protease-resistance core, reflecting the different conformation of tau in tauopathies (15, 51, 52). The detection of ubiquitination densities at K311, K317, and K321 on the fibril core indicates that mono- and polyubiquitin chains form a stable and critical component of pathological tau aggregates (15). These findings together with our results point out that tau at MTBR and the C-terminal region are substrates of ubiquitination, which conventionally occurs throughout the tau aggregation process from oligomers to PHF tau.

In tauopathies such as AD, pathological tau aggregate or seed can spread and be uptaken by the connected cells *via* multiple mechanisms (53, 54, 55, 56). The propagation of pathological tau is prion-like, which refers to the capability of tau seeds to serve as templates to induce further tau assembly and initiate self-amplification (57, 58). In this study, we revealed the relation of ubiquitination and tau seeding cascade in a widely used tau biosensor cell culture (37, 38). The soluble tau aggregates purified from AD brain tissue showed a vast seeding activity, a process previously shown to correlate with clinical severity in AD patients (31, 36, 55). Noticeably, the newly formed tau aggregates directly cooperated with K63-linked ubiquitin, but not K48, which mediates protein degradation by 26S proteasome (14, 59). Our *in vitro* studies in iHEK-Tau cells and Htau-expressing primary neurons also showed that soluble pathological tau aggregate triggered by AD TauO had selective interaction with K63-linked ubiquitin. State-of-the-art SRM-MS analysis demonstrated that K48 and K11-linkages preferentially bind to insoluble tau aggregates rather than soluble tau forms. Strikingly, overexpression of K63-linkages enhanced tau release together with proteasome and autophagy-lysosome dysfunction after AD TauO induction. The selective ubiquitin response on pathological tau forms at early state may be from several possibilities. We postulate that K48-linked ubiquitin, normally found on PHF tau (22), may not be efficient to bind to the newly seeded tau as opposed to K63-linked ubiquitin. Another possibility is that K63-linked ubiquitin preferentially binds to tau oligomers compared with K48-linked ubiquitin. Alternatively, the misfolded aggregates are sent to autophagic structures *via* cargo receptors, such as p62/SQSTM1, which contain both ubiquitin-binding domains and LC3-interating region (60, 61). This selective autophagy can function on individual misfolded protein or on higher-order oligomers or aggregates such as tau (17, 55, 60, 61). For instance, the second abundant K63-linked ubiquitin can signal ALP recognition on tau *via* E3 ligase TNF receptor-associated factor 6 (14, 17, 39). Moreover, hyperactivation of p300/CBP *via* ALP promotes tau secretion and propagation (62), referring to our results that K63-linkage overexpression may trigger tau release through ALP dysfunction. In addition, roles of K63-linked ubiquitin are not limited to the regulation of protein degradation. The specific ESCRT0 components, including Hrs and STAM, have been reported to prevent the binding of 26S proteasome to the K63-conjugated E3, suggesting the role of K63-linked ubiquitin on endosomal-lysosomal pathway (63). Even though, WT-ubiquitin overexpressing cells showed more tau accumulation in cytosolic and nuclear fractions, these aggregates might be processed for cellular degradation. On the contrary, less tau accumulation in K63-ubiquitin overexpressing cells might be associated with increased release of tau as detected from the cultured media. K63-linked tau aggregates are relatively less prone to proteolysis (64), thus allowing them to be available for release and propagation. Together, our findings indicate that the newly seeded tau aggregates may be able to avoid the K48-linkage-mediated proteasome degradation by preferential K63-linked ubiquitination. Another possibility could be that specific ubiquitin linkage determines the fate of tau oligomers as whether they will be directed for proteasomal degradation or will avoid cellular degradation pathway to sustain in the system and exert their toxic effects.

To get insight into the signaling mechanisms regarding K63-linkage-mediated pathological tau formation and secretion by AD TauO, we found highly activated expression of HSP27, p38, p53, and BAD protein, which are responsible for cellular protein homeostasis, cellular stress, and apoptosis. Heat shock protein (HSP) plays a key role in cellular protein homeostasis by preventing the aggregation of partially unfolded protein and maintaining a soluble, folding-competent state (65). HSP27 is a broadly expressed member of the small HSPs and has been implicated in neurodegeneration including AD (66, 67). Here, we found the elevated HSP27 levels correlated with AD TauO and K63-linkage overexpression, indicating a compensation mechanism of HSP/chaperones that is reported to occur at this early state to reduce pathological tau aggregate formation (68, 69). The proapoptotic BAD and P38 MAPK, which have been implicated with oligomeric amyloid-β or tau-induced neuronal cell death (70, 71, 72), were observed in AD TauO and K63-linkage-mediated tau pathology in our present study. Recently, results showed that higher levels of p53 levels were higher after AD TauO treatment, suggesting that the interaction of p53 with TauO impairs DNA damage response (73). Concordantly, the proapoptotic BAD and P38 MAPK, implicated in oligomeric amyloid-β or tau-induced neuronal cell death (70, 71, 72), were also observed in AD TauO and K63-linkage-mediated tau pathology in our present study. These observations suggest that AD TauO-treated cells will subsequently undergo apoptotic pathway.

Besides, the activation of EGFR, HDAC4, SMAD1, and SMAD2 were observed. Previous studies suggest that the polymorphisms of *EFGR* gene associate with an increased risk of AD and several human diseases (66, 67). Histone deacetylase 4 (HDAC4) and TGFβ/Smad pathway are actively involved in regulating the transcription of genes involved in synaptic plasticity and neuronal survival by interacting with transcription factors (43, 74, 75). We found that activated HDAC4, Smad 1, and Smad2 levels were observed in our *in vitro* study. The possibility of this alternation may be due to the inhibition of neurogenesis (76), the activation of ER stress (77), and neuronal cell death (78) associated with HDAC4 upregulation (79). The activation of Smad signaling by tau aggregates has been reported that may adversely affect the survival of neurons in AD (80, 81). However, future studies will be needed to clearly dissect this possibility.

We have found that AD tau oligomers promote selective K63-linked ubiquitination, which results in enhanced soluble tau formation with increased tau secretion (Fig. 7). Accordingly, several underlying mechanisms associated with protein homeostasis, cellular stress, and apoptotic cell death were shown in this study that relate to the AD-liked pathology. Therefore, our study highlights the significance of selective ubiquitination of tau oligomers in pathological propagation. The insight from this study holds great promise for targeted therapeutic intervention in AD and related tauopathies.

## Experimental procedures

### Brain homogenate preparation

Postmortem brain tissue samples from AD and control subjects were obtained from Oregon Health and Science University, the Institute for Brain Aging and Dementia (University of California–Irvine, Irvine, California, USA), and the Brain Resource Center at Johns Hopkins and approved by the Institutional Ethics Committee. Neuropathological assessment conformed to National Institute on Aging/Reagan Institute consensus criteria. The following information was available for the cases used in this study: diagnosis, age at death, gender, post-mortem index, brain area, and Braak stage (Table S3).

### Separation of soluble and insoluble tau

Separation of PBS soluble and insoluble tau was performed in accordance with protocols previously described (23). In brief, brain tissues and cell lysates were homogenized in 1X phosphate-buffered saline (PBS) with protease inhibitor (Roche) at 1:3 (w/v) ratio and incubated for 20 min at 4 °C. Then the samples were centrifuged at 11,000*g* for 20 min at 4 °C. The supernatant was collected and centrifuged at 100,000*g* for 60 min at 4 °C. To extract the PBS insoluble tau, the pellets from the first and second cold centrifugation were combined and resuspended in PHF extraction buffer (10 mM Tris-HCl.1 mM EGTA, 0.85 M NaCl, 10% (w/v) sucrose, pH 7.4) at 1:10 (w/v) ratio. Samples were centrifuged at 15,000*g* for 20 min at 4 °C. Sarkosyl (SRK) solution was added to the supernatant at a final concentration of 1% and stirred for 1 h at room temperature (RT) prior to centrifugation at 100,000*g* for 30 min at 4 °C. Finally, the SRK insoluble fraction was made by resuspending the pellet in 1X PBS depending on the amount of starting material (1 ml buffer for 25 g of starting material). The total protein concentrations of the final fractions were analyzed by bicinchoninic acid (BCA) assay (Thermo Scientific).

### Immunoprecipitation (IP)

Samples from human brain homogenates or cell lysates were immunoprecipitated with a misfolded tau specific T18 antibody (31), K63-likage specific polyubiquitin antibody (Cell Signaling Technology; 5621), and K48-likage specific polyubiquitin antibody (Cell Signaling Technology; 8081) using Pierce Co-Immunoprecipitation Kit (Thermo Scientific). Briefly, amine-reactive resin was coupled with affinity-purified T18 antibody, incubated with samples. Bound proteins were eluted in 0.1 M glycine (pH 2.8), the pH was adjusted to 7.0 by adding 1 M Tris-HCl (pH 8). Isolated fractions were subjected to buffer exchange against 1X PBS followed by Western blot analysis. The total protein concentration was measured with a BCA assay.

### Tau biosensor cell culture and seeding assay

Tau biosensor cells (ATCC; CRL-3275) were cultured in DMEM supplemented with 10% FBS, 100 μg/ml penicillin, and 100 μg/ml streptomycin. Cell cultures were maintained in a humidified atmosphere equipped with 5% CO_2_ at 37 °C. To determine the seeding activity of PBS- or SRK-soluble tau, cells were plated on poly-L-lysine-coated coverslips at a density of 1 × 10^5^ cells/well in 24-well plates. Cells were then exposed to tau aggregates (0.01 and 0.05 μM) in the presence of Lipofectamine 2000 (Invitrogen) for 24 h followed by three washes with 1X PBS. Coverslips were fixed with 4% formaldehyde and mounted with Prolong Gold mounting media for imaging. Each condition for this assay was performed in triplicates.

### iHEK-Tau cell culture, transfection, and treatment

Tetracycline (Tet)-regulated human WT tau 4R0N stable cell line was generated as described previously (82). Cells were cultured in Dulbecco’s modified Eagle’s medium (DMEM) supplemented with 10% fetal bovine serum (Gibco), 100 U/ml penicillin G, 250 ng/ml amphotericin B, 100 μg/ml streptomycin. Culture conditions were maintained at 37 °C in a humidified atmosphere containing 5% CO_2_. For DNA plasmid transfection experiments, plasmids expressing HA-tagged ubiquitin WT and mutants were generated and provided by Prof. Lim Kah Leong as described elsewhere (17). Cells were transiently transfected with an empirical concentration (250 ng) of HA-tagged ubiquitin plasmids (WT, K48, K48R, K63, K63R) for 24 h using Lipofectamine 2000 (Invitrogen) according to manufacturer’s instructions. To induce tau expression, cells were treated with Tet (1 μg/ml, Sigma) for 24 h. For oligomeric tau treatment, cells were incubated with 0.1 μM AD brain-derived tau oligomers (55) (AD TauO) for 3 h. The media was collected and replaced with fresh media. Cells were separated soluble and insoluble tau and characterized after 2 days by Western blot. Conditioned media was centrifuged at 400*g* for 5 min at 4 °C. Supernatants were concentrated using 10k Amicon Ultra-0.5 centrifugal filter (Millipore) and centrifuged at 3000*g* for 60 min at 4 °C. The filtrates were collected in fresh tubes before LC-MS/MS (83) and Western blot analysis. Preparation and characterization of AD TauO were previously published from our laboratory and explained more detail in Supplementary Information.

### Cell fractionation

iHEK-Tau cells (5 × 10^5^ cells/ml) were cultured in a 6-well plate. After transfection and AD TauO treatment, cells were fractionated using the Qproteome cell compartment kit (QIAGEN; 37502). Briefly, cells were scraped, resuspended in ice-cold lysis buffer, and centrifuged at 1000*g* for 10 min. Supernatant (cytosolic fraction) was collected. Pellets were further incubated in extraction buffer CE2 for 30 min at 4 °C followed by spinning at 6000*g* for 10 min. Supernatant was discarded, then pellets were further extracted with Benzonase nuclease for nuclear fraction. Proteins from fractionations were quantified using Pierce BCA protein assay kit (Thermo Scientific).

### Primary neuron preparation

Primary cortical neuronal cultures were prepared and maintained as described previously (55). Briefly, cortical neurons were isolated from human tau (Htau)-expressing mice (Jackson Laboratory; 005491) during embryonic day 13 to 16 using Accutase solution (Sigma) together with gentle trituration by a fire-polished glass pasture pipet. Dissociated cells were plated at a density of 1.6 × 10^5^ cells/ml on poly-L-lysine coated coverslips or 2.5 × 10^5^ cells/ml in 6-well plates. Culture media contains neurobasal medium (Gibco; 12348017) supplemented with 2% B-27 Plus supplement (Gibco; A3582801), 0.5 mM GlutaMax (Gibco; 35050–061), 10,000 units/ml penicillin, 10,000 μg/ml streptomycin, and 25 μg/ml amphotericin B (Gibco; 15240062). Half of the media was changed every 3 to 4 days. Cells on 10 to 13 days *in vitro* (DIV) were used for all experiments.

### Mass spectrometry

#### Trypsin digestion of tau aggregates

The detergent that remained in IP tau preparation was removed by a microcentrifuge filter unit (molecular cutoff 30 kDa) (Millipore). Ten micrograms of tau samples was added into a filter unit, respectively. Then 200 μl of 25 mM ammonium bicarbonate (pH 8.0) was added into each filter unit and centrifuged at 12,000*g* for 10 min. This step was repeated twice. The remained tau samples in the filter were transferred into a 0.6-ml tube, and 0.2 μg of trypsin was added to each sample and incubated at 37 °C overnight. The samples were then desalted with ZipTip C18 before mass spectrometry analysis.

#### LC-MS/MS analysis of total tau and ubiquitinated tau

For LC-MS analyses, the peptides were analyzed with Easy nLC1000 UHPLC-Q Exactive Orbitrap LC-MS system (Thermo Scientific). A 1-h linear gradient from 2% solvent A (0.1% formic acid in water) to 35% solvent B (0.1% formic acid in acetonitrile) was used for each LC-MS/MS run. The resolution of the full scan was 70,000 (@m/z 200), the target AGC value was set to three × 10^6^, and maximum fill time was 200 ms for the full scan; 17,500 (@m/z 200), a target AGC value of two × 10^5^, and maximum fill times of 100 ms for MS2 scan. Mass spectra were analyzed using MaxQuant software (version 1.5.2.8) (84). The initial maximum allowed mass deviation was set to 10 ppm for monoisotopic precursor ions and 0.5 Da for MS/MS peaks. Enzyme specificity was set to trypsin, defined as C-terminal to arginine and lysine excluding proline, and a maximum of two missed cleavages were allowed. Carbamidomethylcysteine was set as a fixed modification and methionine oxidation, acetylation, and ubiquitination of lysine as variable modifications. The spectra were searched by the Andromeda search engine against the Human SWISSPORT sequence database (containing 20,193 human protein entries) combined with 248 common contaminants, and concatenated with the reversed versions of all sequences. The required false-positive rate for identification was set to 1% at the peptide level and 1% at the protein level, and the minimum required peptide length was set to 6 amino acids. Contaminants, reverse identification, and proteins only identified by modified peptides were excluded from further data analysis.

#### Stable isotope dilution-SRM-MS analysis of ubiquitination linkages

The SID-SRM-MS assays of selected proteins were developed as described previously (85). For protein tau, two or three peptides were initially selected, and then the sensitivity and selectivity of these were experimentally evaluated as described previously (85). The peptide with the best sensitivity and selectivity was selected as the surrogate for that protein. For each peptide, 3–5 SRM transitions were monitored (Table S2). The signature peptides of various ubiquitination linkages were chemically synthesized, incorporating isotopically labeled [^13^C_6_^15^N_4_] arginine or [^13^C_6_^15^N_2_] lysine to a 99% isotopic enrichment (Thermo Scientific). The amount of stable isotope-labeled standard (SIS) peptides was determined by amino acid analysis. After trypsin digestion, the tau peptides were then reconstituted in 30 μl of 5% formic acid-0.01% TFA, and an aliquot of SIS peptides was added to each tryptic digest. These samples were desalted with a ZipTip C18 cartridge and analyzed by LC-SRM-MS as described above.

#### *In situ* proximity ligation assay (PLA)

To identify the interaction of oligomeric tau and ubiquitin in postmortem human brain tissues, the Duolink *In Situ* Red starter kit mouse/rabbit (Sigma Aldrich; DUO92101) was used according to the manufacturer’s guideline. Briefly, frozen human brain tissues were pretreated with chilled methanol prior to incubate with blocking solution at 37 °C. After 1 h, primary antibody solution was added overnight at 4 °C. Primary antibodies used include rabbit anti-T22 (1:200) and mouse anti-ubiquitinylated protein, clone FK2 (1:500, Sigma-Aldrich; 04–263), After two washes for 5 min with 1X wash buffer A, PLA probe solution was applied for 1 h at 37 °C. Tissues were washed and incubated with ligation buffer for 30 min at 37 °C followed by washing and adding the amplification solution for 100 min at 37 °C. Slides were then washed 10 min twice with 1X washing buffer B at RT before mounting using Duolink *In Situ* Mounting Medium with DAPI.

#### Immunofluorescence of human and mouse brain tissues

Immunofluorescence assays were performed with frozen sections of frontal cortices from human and mouse brains. The sections were fixed in chilled methanol followed by blocking in blocking buffer (5% BSA and 5% normal goat serum in 1X PBS supplemented with 0.25% Triton X-100 (PBST)) for 2 h at RT. Sections were then incubated in primary antibodies diluted in 5% BSA in PBST for overnight at 4 °C, including rabbit anti-T18 (1:500), chicken anti-Total Tau (1:1000, Abcam; ab75714), rabbit anti-ubiquitin (linkage-specific K48) (1:500, Abcam; ab140601), rabbit anti-ubiquitin (linkage-specific K63) (1:500, Abcam; ab179434), mouse anti-ubiquitinylated protein, clone FK2 (1:500, Sigma-Aldrich; 04–263), mouse anti-βIII-tubulin (1:1000, Abcam; ab78078), chicken anti-GFAP (1:1000, Abcam; ab4674), or guinea pig anti-IBA1 (1:500, Synaptic Systems; 234,004). After washing three times with 1X PBS (10 min each), sections were incubated in Alexa-conjugated antibodies (1:700, Invitrogen) diluted in 5% BSA in PBST for 2 h at RT. Slides were washed and mounted using Prolong Gold antifade reagent with DAPI (Invitrogen, P36935).

#### Immunocytochemistry

Tau biosensor cells or primary neurons were grown on poly-L-lysine-coated coverslips. After cell treatments, cells were gently washed three times with 1X PBS. Formaldehyde solution 4% (Sigma) was used for fixation for 15 min at RT followed by three washes. Cells were permeabilized using 0.25% Triton X-100 (Sigma) in 1X PBS for 10 min and blocked for 30 min at RT in blocking buffer. Cells were then incubated with primary antibodies diluted in blocking buffer overnight at 4 °C. Primary antibodies used include rabbit anti-ubiquitin (linkage-specific K48) (1:500, Abcam; ab140601), rabbit anti-ubiquitin (linkage-specific K63) (1:500, Abcam; ab179434), and mouse anti-toxic tau conformation specific TTCM1 antibody. On the next day, cells were washed and incubated with Alexa-conjugated anti-rabbit IgG antibodies (1:1000, Life Technologies) for 1 h at RT in the dark. After three washes, cells were mounted with Prolong Gold antifade reagent with DAPI.

#### Image analysis

Brain sections or cells were imaged with 63x objective of a Zeiss LSM 880 with Airyscan confocal microscope sing 405 nm blue diode, 458/488/514 nm Argon, and 633 nm He 633 lasers. To build the z-stack, 17 stacks/0.37–0.41-μm optimal thickness was captured. Each brain or cell slide was randomly imaged in 5 to 6 different regions of interest in duplicate. All images were analyzed *via* Pearson’s correlation coefficient (PCC) for protein colocalization using ImageJ (NIH) and Imaris software (Bitplane).

#### Proteasome activity assay

iHEK-Tau cells were processed for proteasome activity according to published methods (10, 86). Briefly, cells were homogenized in a proteasome lysis/assay buffer (50 mM Tris-HCl, pH 7.4, 5 mM MgCl_2_, 5 mM ATP, 1 mM DTT, 10% glycerol) and further lysed by passing 15 times through a 29-guage needle. Lysates were centrifuged at 20,000*g* for 20 min at 4 °C. Supernatant was quantified for protein concentration using Bradford protein assay (Biorad). Ten micrograms of total protein was diluted with proteasome assay buffer and incubated with Suc-LLVY-AMC (Bachem; 94367–21–2). AMC released fluorescence was monitored on fluorescent plate reader at A_360_ex/A_460_em every 10 min for 1 h at 37 °C.

#### Multi-pathway profiling assay

iHEK-Tau cells were evaluated for cell signaling pathway alteration caused by AD TauO treatment using the Human Phosphorylation Multi-Profiling Array C55 (RayBiotech; AAH-PPP-1-4) (87), following the manufacturer’s instructions. Briefly, membranes were incubated with blocking buffer at RT for 30 min, and cell lysate (30 μg) was applied overnight at 4 °C. Membranes were washed triple or twice with wash buffer I and II, respectively, then incubated with detection antibody cocktail overnight at 4 °C. After washing with wash buffers I and II, membranes were incubated with horseradish peroxidase (HRP)-labeled anti-rabbit IgG for 2 h at RT. The signals were detected by X-ray films after applying electrochemiluminescent reagent. The intensities of array dots were quantified in quadruplet per array with ImageJ software (NIH) and were normalized against the positive controls on the blots.

#### Western blot and dot blot analysis

For Western blot analysis, equal proportion of PBS- or SRK-soluble fractions of IP tau isolated from human brain homogenate, mouse hippocampal homogenate, or cells was loaded on precast NuPAGE 4 to 12% Bis-Tris gels (Invitrogen) for SDS-PAGE analysis. Gels were subsequently transferred onto nitrocellulose membranes and blocked for 1 h at RT using Odyssey Blocking Buffer (LI-COR). For dot blot, an equal amount of concentrated cultured media was dotted on the membrane and let dry for 1 h at RT followed by blocking. Membranes were then probed overnight at 4 °C using the corresponding primary antibodies followed by IRdye secondaries (LI-COR) at 1:10,000 for 1 h at RT. Images were acquired by LI-COR Odyssey imager. Densitometric analysis was performed using ImageJ software (NIH).The primary antibodies used were the following; mouse anti-Tau 13 (1:1000, BioLegend; MMS-520R), rabbit anti-Tau (1:5000, Abcam; ab64193), rabbit anti-T18 (1:500), rabbit anti-HA tag (1:1000, Abcam; ab9110), rabbit ubiquitin (linkage-specific K48) (1:5000, Abcam; ab140601), rabbit anti-ubiquitin (linkage-specific K63) (1:5000, Abcam; ab179434), mouse anti-polyubiquitinated protein, clone FK1 (1:1000, Enzo; BML-PW8805), mouse anti-ubiquitinylated protein, clone FK2 (1:5000, Sigma-Aldrich; 04–263), rabbit ubiquitin (1:5000, Abcam; ab7780), rabbit anti-Lamin B1 (1:1000, Abcam; ab16048), rabbit anti-β-actin (1:5000, Abcam; ab8227), and rabbit anti-GAPDH antibody (1:1000, Abcam; ab9485).

#### Statistical analysis

All experiments were repeated at least three times. Statistical analyses were performed using Prism 9.0 (GraphPad Software) through unpaired two-tailed Student’s *t* test or one-way analysis of variance (ANOVA) according to group number. Results are considered statistically significant at *p* < 0.05.

## Data availability

All data are contained within this manuscript and supporting information. The datasets used during the current study are available from the corresponding author on reasonable request. The mass spectrometry proteomics data have been deposited to the ProteomeXchange Consortium *via* the PRIDE partner repository with the dataset identifier PXD031417. SRM-MS data have been deposited to PASSEL with the dataset identifier PASS01732.

## Supporting information

This article contains supporting information (23, 31, 55, 88, 89, 90, 91).

## Conflict of interests

The authors declare that they have no conflict of interests with the contents of this article.
