# Supplementary material for: Lysine 63-linked ubiquitination of tau oligomers contributes to the pathogenesis of Alzheimer’s disease
Source: J Biol Chem. 2022 Feb 22;298(4):101766. doi: 10.1016/j.jbc.2022.101766 (PMC8942844; doi:10.1016/j.jbc.2022.101766)
Supplement: Supplemental Figures S1–S6 [file mmc1.docx]

**Supplementary Information**

**Lysine 63-linked ubiquitination of tau oligomers contributes to the pathogenesis of Alzheimer’s disease**

Nicha Puangmalai^1, 2^, Urmi Sengupta^1, 2^, Nemil Bhatt^1, 2^, Sagar Gaikwad^1, 2^, Mauro Montalbano^1,2^, Arijit Bhuyan^5^, Stephanie Garcia^6^, Salome McAllen^7^, Minal Sonawane^1, 2^, Cynthia Jerez^1, 2^, Yingxin Zhao^3, 4^ and Rakez Kayed^1, 2,^ ^†^

**^1^**Mitchell Center for Neurodegenerative Diseases, University of Texas Medical Branch, Galveston, TX, 77555, USA

**^2^**Departments of Neurology, Neuroscience and Cell Biology, University of Texas Medical Branch, Galveston, TX, 77555, USA

^3^Department of Internal Medicine, University of Texas Medical Branch, Galveston, TX, 77555, USA

^4^Institute for Translational Sciences, University of Texas Medical Branch, Galveston, TX, 77555, USA

^5^School of Medicine, University of Texas Medical Branch, Galveston, TX, 77555, USA

^6^University of Texas Health Science Center, Houston, TX, 77030, USA

^7^University of Texas MD Anderson Cancer Center, Houston, TX, 77030, USA

**† To whom correspondence should be addressed**

**Corresponding Author**

Rakez Kayed, PhD

University of Texas Medical Branch

Medical Research Building Room 10.138C

301 University Blvd

Galveston, TX 77555-1045

Phone: 409.772.0138

Fax: 409.747.0015

e-mail: [rakayed@utmb.edu](mailto:rakayed@utmb.edu)

**Material included:** Supplementary materials and methods, Figure S1-S6.

**Supplementary Materials and methods**

**1. Characterization of brain-derived tau oligomers**

The characterization was performed by various methods as previously described [1-3]. The morphology of immunoprecipitated structures was assessed as previously described by Atomic Force Microscopy (AFM) using a non-contact tapping method (ScanAsyst-air) with a Multimode 8 AFM machine (Veeco, CA). The size was determined by Western blot using Tau-5 antibody. Size-exclusion chromatography analysis and purifications were performed using a LC-6AD Shimadsu high-performance liquid chromatography (HPLC) system fitted with a Superdex200 increase (30 cm x 10 mm) column, Supelco-808541. PBS, pH 7.4, was used as the mobile phase, flow rate 0.5 ml/min. Gel filtration standard (Bio-Rad; 51-1901) was used for calibrations. Thioflavin T (ThT) assay**:** samples were prepared using 1 μl of protein sample and 250 μl of 5 μM ThT, 50 mM glycine-NaOH buffer (pH 8.5) in clear bottom 96-well black plates. The ThT fluorescence intensity of each sample was recorded using a POLARstar OMEGA plate reader (BMG Labtechnologies, Melbourne, VIC, Australia) with Fluorescence was measured at λ-emission 490 nm upon excitation at λ-excitation 440 nm. For the Bis-ANS assay, samples were prepared using 1 μl of protein sample and 250 μl of 10 μM Bis-ANS, 100 mM glycine-NaOH buffer (pH 7.4) in clear bottom 96-well black plates. The ThT fluorescence intensity of each sample was recorded using a POLARstar OMEGA plate reader (BMG Labtechnologies, Melbourne, VIC, Australia) with λ-emission 520 nm upon excitation at λ-excitation 380 nm. Western blot analysis was performed as we recently described [4], PBS-soluble fractions of brain extracts were run on Bis-Tris SDS-PAGE gels and subsequently transferred onto nitrocellulose. After blocking overnight at 4°C with 10% nonfat dried milk, membranes were probed for 1 h at room temperature with anti-tau oligomer antibody T22 [5].

**2. Preparation of recombinant tau species**

Recombinant tau protein [tau-441 (2N4R) MW 45.9 kDa] was expressed and purified as described previously [6]. Tau protein was treated with 8 M urea to obtain monomeric tau and dialyzed overnight against molecular grade water (pH 7.4). Tau was aliquoted and lyophilized. For preparation of oligomers, lyophilized tau was resuspended in 1 ml of 1X PBS and diluted to make a final concentration of 0.3 mg/ml. Aliquots of a monomeric tau solution were prepared in sterile water with 1×PBS and incubated with Heparin (15 kDa) in a 1:4 ratio of Heparin to TauM [5] at 37°C for 3–5 days. The tau-Heparin mixture was rotated constantly using a rotary shaker at a speed of 30 rpm. The characterization of tau oligomers and fibrils was performed as described previously [1].

**3. Seeding Assay**

Recombinant tau 4R monomers were obtained by dissolving lyophilized pellets of recombinant 4R tau at 1 mg/mL concentration in PBS [7] and seeded with AD-brain derived tau oligomers. The oligomer-monomer mixture was made at a ratio of 1:100 (w/w) with gentle agitation at room temperature for 2 days. After seeding, aliquots were taken and immediately used for Western blotting using T22 and Tau-13 antibody as well as AFM analysis. Total protein concentration was determined using the BCA assay and stored at -20°C until use.

**Supplementary Figures
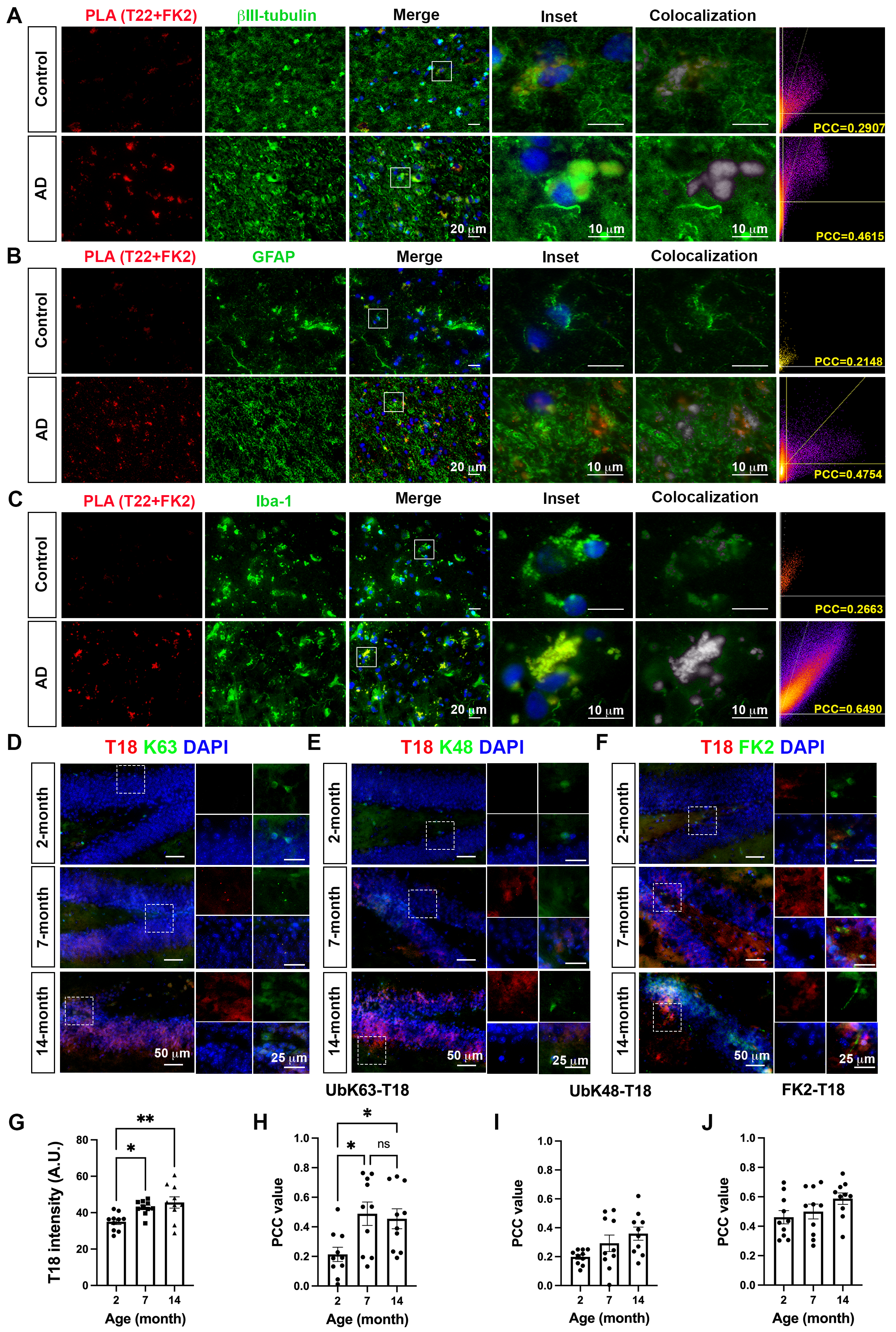
**

**Figure S1. Ubiquitinated tau oligomers observed in neuronal and glial cells in AD brain tissues and transgenic mouse model of tauopathy, Related to Fig. 1.**

**A-C** Representative images of AD human brain tissues showed an increased, direct interaction of tau oligomers with ubiquitin (PLA, red) compared to control as well as colocalization with (**A**) mature neurons (βIII-tubulin, green), (**B**) astrocytes (GFAP, green), and (**C**) microglial cells (Iba-1, green). Scale bar = 20 μm. Insets represent high magnification of ROIs in rectangles, together with colocalization map and PCC analysis.

**D-G** Representative immunofluorescence images of the dentate gyrus of 2-mo, 7-mo, and 14-mo-old Htau (N = 3) showed increased expression of misfolded tau (T18, red).

**H-J** PCC analysis showed significantly higher colocalization of T18-positive tau with (**D**, **H**) K63-ubiquitin chains (K63, green) in age-dependent manner, but not with (**E, I**) K48-ubiquitin chains (K48, green) nor (**F, J**) FK2-positive ubiquitin (FK2, green). Scale bar = 50 µm. ROIs in rectangles showed in separate channels on right panels. Analysis of T18 intensity and PCC were measured and showed as mean ± SD. Statistical analyses were calculated by One-way ANOVA, followed by Tukey’s test. (**p* < 0.05, ***p* < 0.01).

**
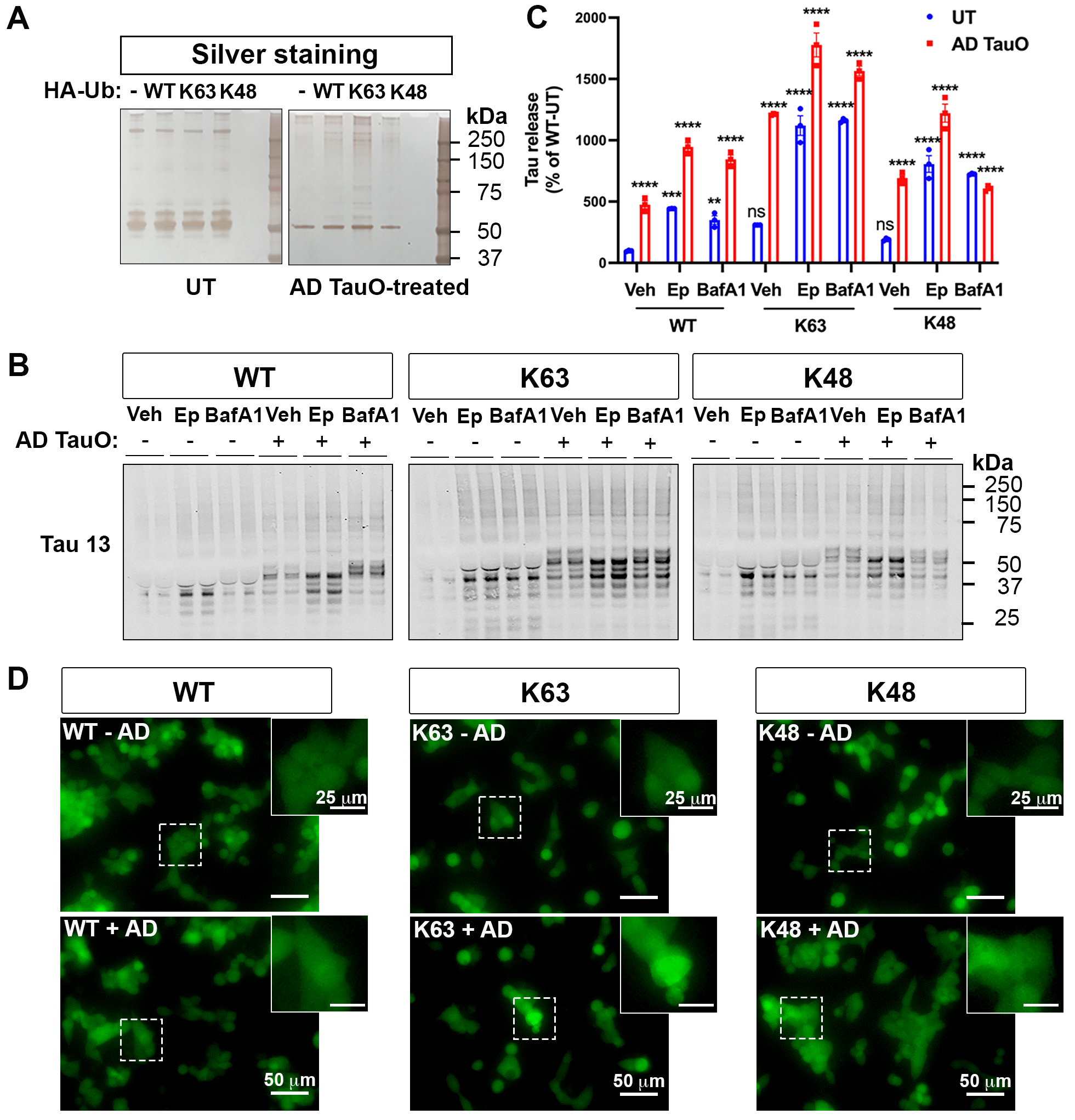
Figure S2. K63-linkage linked to extracellular protein release in AD TauO-induced iHEK-Tau cells, Related to Fig. 4.**

**A** AD TauO triggered extracellular protein secretion. Total protein detected by silver staining of concentrated cultured media from experimental design in **Fig. 4A**.

**B, C** Diverse influences of ubiquitin isoforms on tau release. WT-, K63-, and K48 ubiquitin chains-transfected iHEK-tau cells were prepared and treated as shown in experimental design in **Fig 4A**. At 24 h prior to sample collection, cells were incubated with 0.1 μM epoxomicin (Ep), or Bafilomicin A1 (BafA1). 0.01% DMSO was used as vehicle (Veh). Immunoblot analysis of total tau (Tau 13) from concentrated media was measured and showed as mean ± SD. Statistical analyses were calculated by One-way ANOVA, followed by Tukey’s test. (*****p* < 0.0001 compared to untreated WT ubiquitin-expressing cells).

**D** Released tau from K63-linkage overexpressing cells contain seeding activity. Representative live imaging after 72 h of Tau biosensor cells treated with concentrated media from WT, K63 or K48-linkage overexpressing iHEK-Tau cells with or without AD TauO exposure as mentioned in **Fig 4A**. Scale bar = 50 μm. ROIs in rectangles showed at 2X digital zoom on top right corners.

**
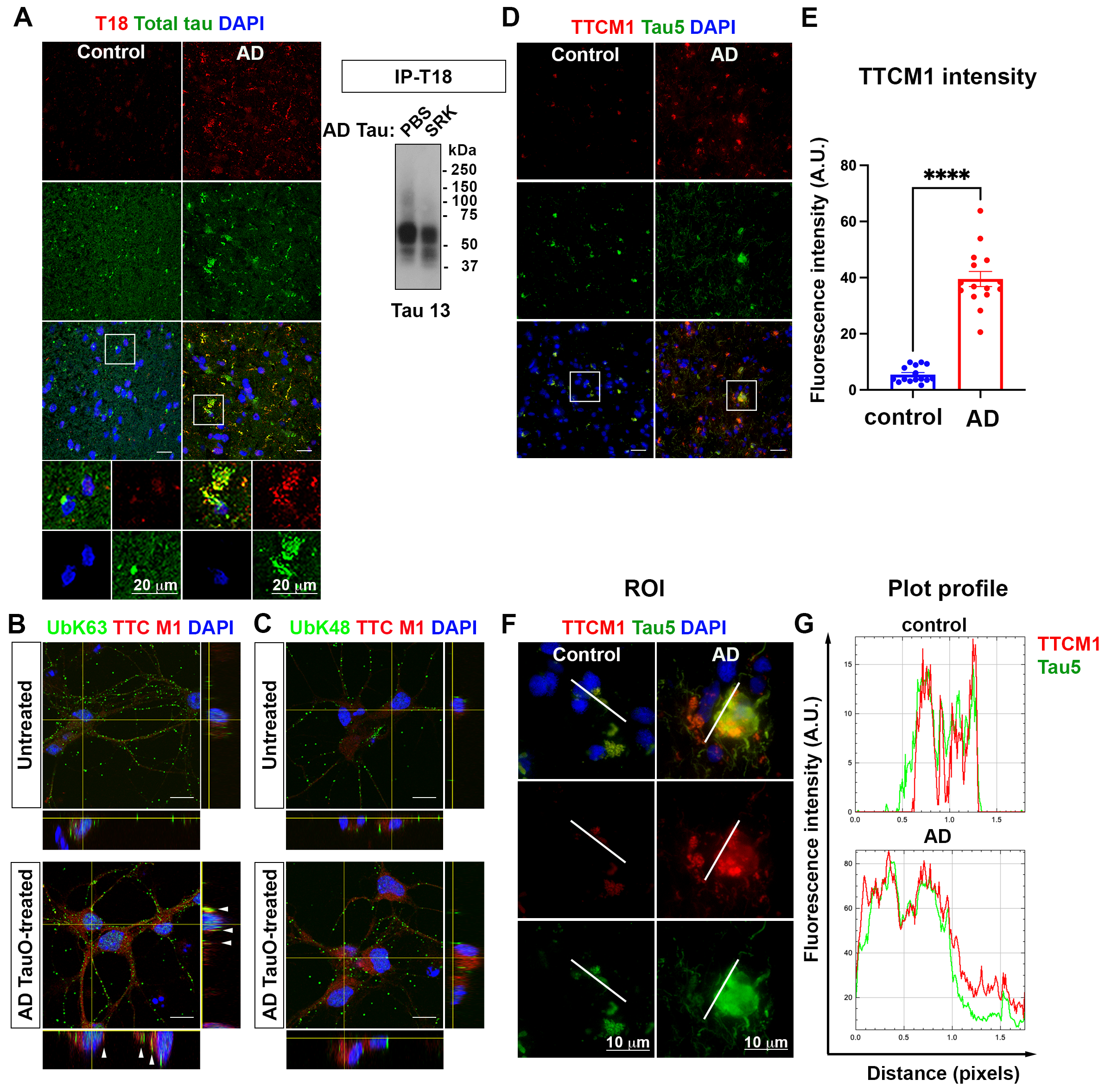
Figure S3. Immunofluorescence of toxic tau aggregates recognized by T18 and TTCM1 antibodies in AD human brain tissues, Related to Fig. 1, 2, and 5.**

**A** Representative confocal images showed toxic tau aggregates (T18, red) and total tau (Total tau, green), merged with nuclei (DAPI, blue), in human AD and control brain tissues (N = 3 cases). Scale bar = 20 µm. ROIs are outlined in white and showed at 2X digital zoom in separate and combined color channels. Co-localization of T18 and total tau is represented in yellow. Representative immunolabeling of T18 immunoprecipitated AD tau aggregates using Tau 13 antibody showed high molecular weight tau aggregates in PBS-soluble compared to SRK-soluble fractions.

**B, C** Immunofluorescence reveals K63-linked ubiquitin colocalization with pathological tau in AD TauO-treated MAPT primary neurons*.* Primary neuronal cultures derived from Htau mice were exposed to AD TauO, followed by immunostaining with **(B)** anti-K63-linked ubiquitin antibody (UbK63, green), **(C)** anti-K48-linked ubiquitin antibody (UbK48, green), and anti-pathological tau antibody (TTCM1, red). DAPI was used for nuclei staining. Representative orthogonal micrographs showed colocalization of pathological tau with K63-linked ubiquitin (arrow heads) after treatment with AD TauO. Scale bar = 20 mm.

**D, E** Representative images of AD brain tissues showed an increase intensity of toxic tau aggregates (TTCM1, red) that colocalized with total tau (Tau 5, green), and merge with nuclei (DAPI, blue) compared to healthy control brains. Scale bar = 20 µm. (**C**) Image analysis showed as mean ± SD. Statistical analyses were calculated by unpaired two-tailed Student’s *t*-test (*****p* < 0.0001).

**F, G** Insets represent high magnification of ROIs in rectangles shown in **D**, together with (**G**) spatial plot profiles of TTCM1 (red) and Tau5 (green) fluorescence intensity.

**
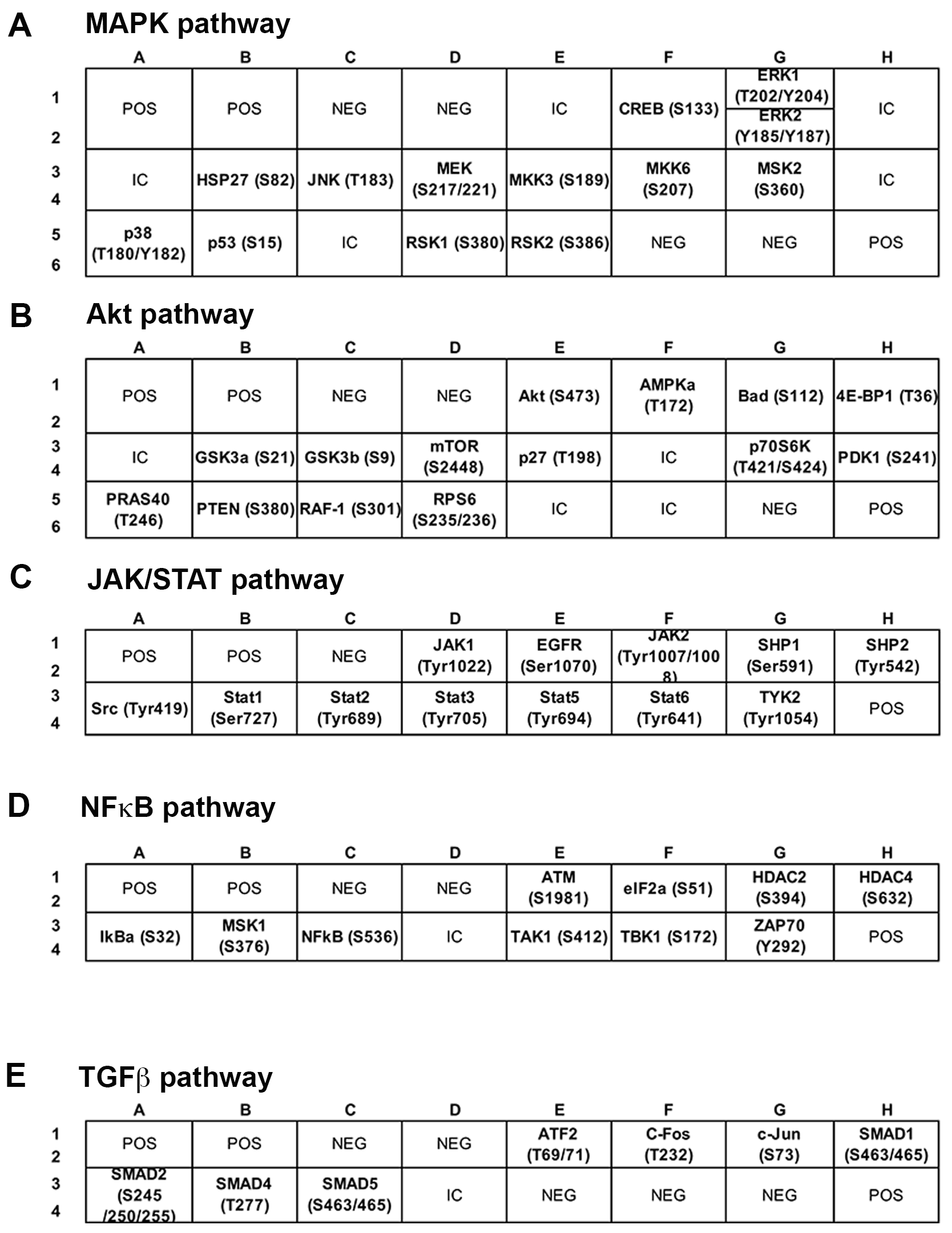
**

**Figure S4. MAPK, Akt, JAK/STAT, NFκB, and TGFβ signaling in AD TauO-treated K63-linkage overexpressing-iHEK Tau cells, Related to Fig. 6.**

**A-E** Signaling pathway profiling maps of (**A**) MAPK, (**B**) Akt, (**C**) JAK/STAT, (**D**) NFκB, and (**E**) TGFβ array. POS = positive control, NEG = negative control, IC = internal control for company uses only.

**
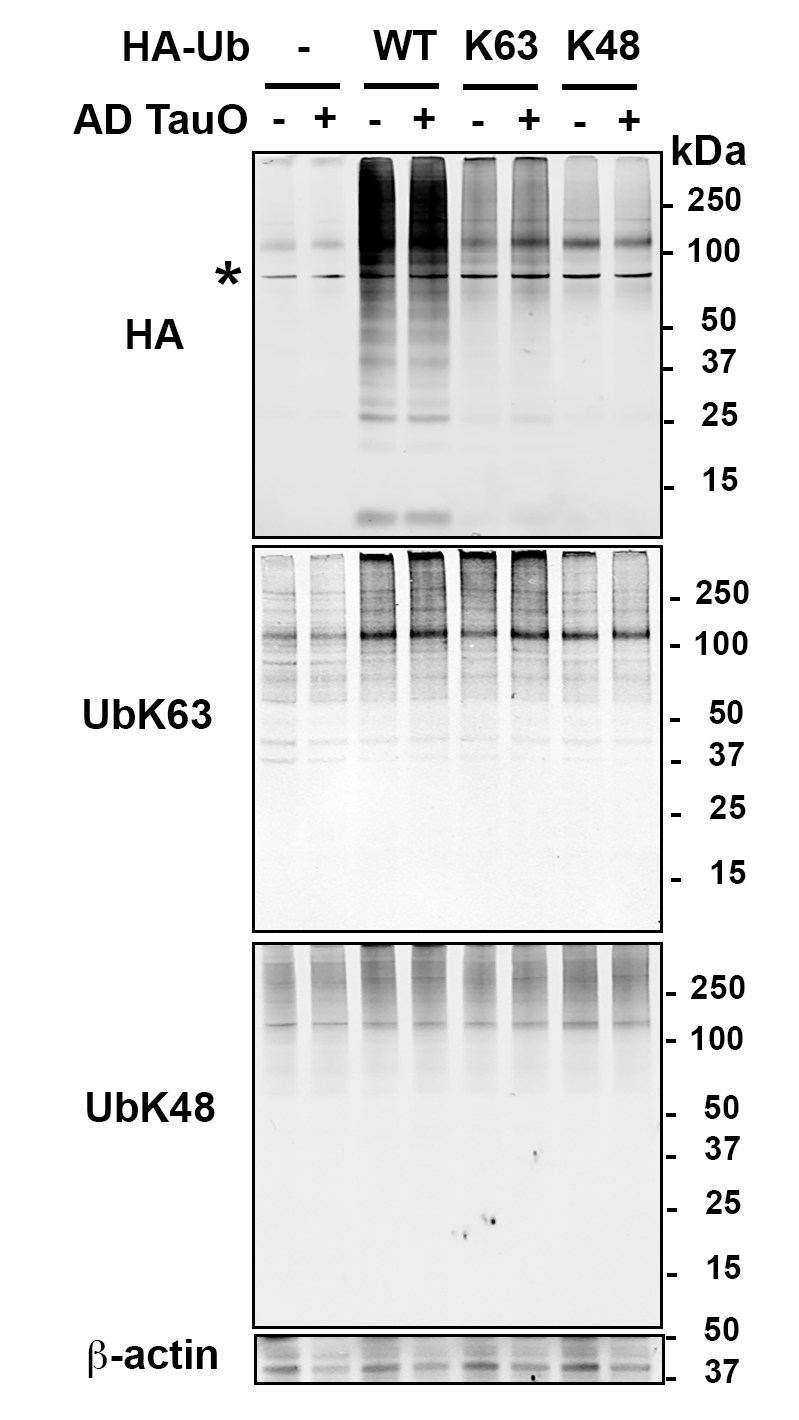
**

**Figure S5. Validation of HA-ubiquitin isoform expression in iHEK-Tau cells**

Representative images of Western blot of total cell lysates from iHEK-Tau cells (n = 3) showed patterns of HA-Ub isoform expression (top). The asterisk represented non-specific binding of anti-HA antibody. Anti-UbK63 (middle) and anti-UbK48 (bottom) antibodies detected both endogenous levels of ubiquitin species and overexpression levels. HA-Ub transfection and AD TauO treatment were mentioned in **Fig. 4A**. β-actin was used as loading control.

**
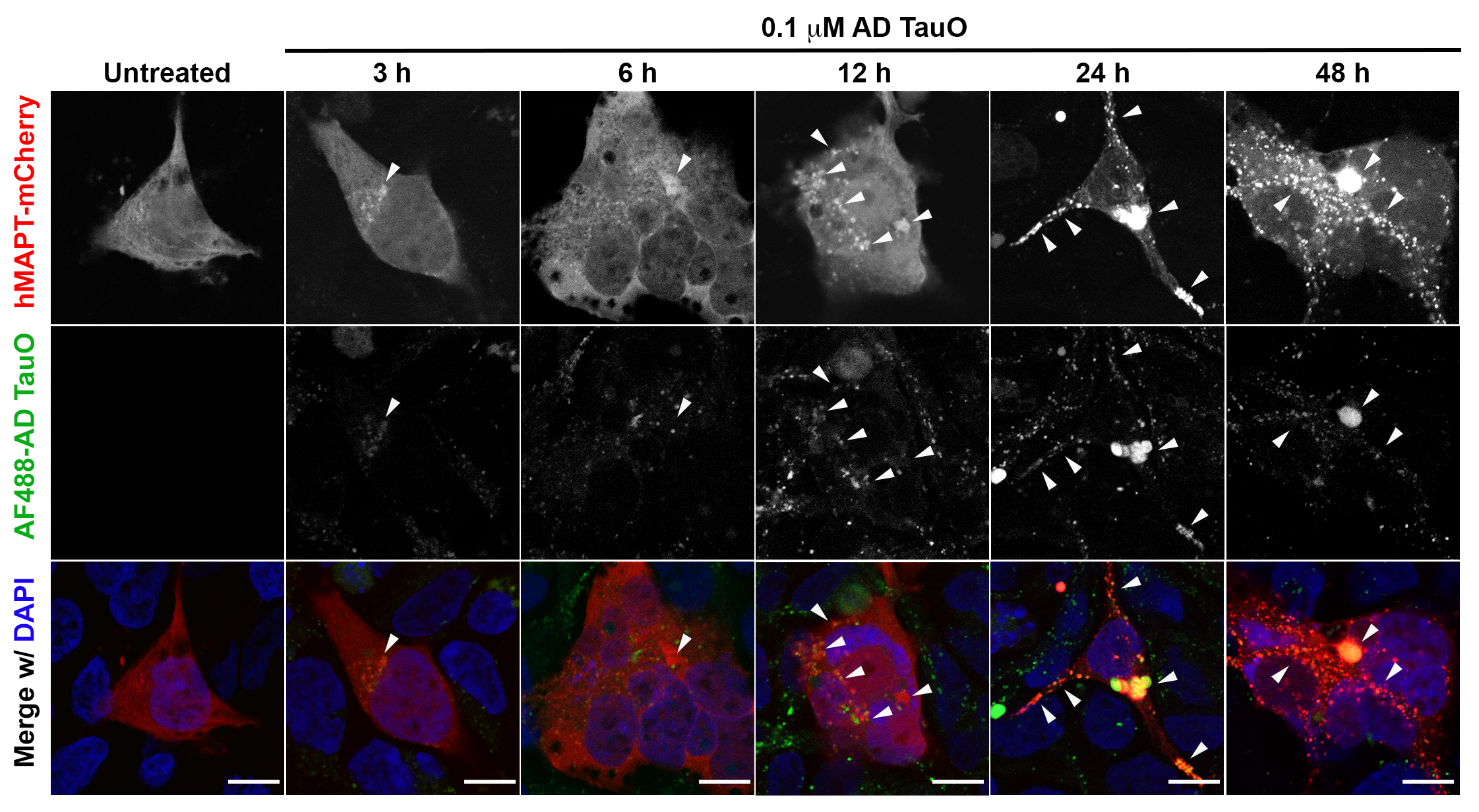
**

**Figure S6.** **AD TauO induced endogenous tau aggregates.**

Representative confocal images of tau aggregate formation in human MAPT (hMAPT)-mCherry tagged overexpressing HEK293T cells after AD TauO treatment in time-dependent manner. hMAPT-mCherry plasmids (pRP[Exp]-mCherry/Puro-CAG>hMAPT, VectorBuilder) (red) were transfected in HEK293T cells for 48 h. Cells were treated with AlexaFluor488 (AF488)-labeled AD TauO (green) for 3 h prior to replace with fresh media. Cells were fixed after 3-48 h followed by confocal imaging. Endogenous tau (red) started forming aggregates (white arrow heads) at 3 h. At 24 and 48 h post-treatment, endogenous tau showed bigger size of tau aggregates in cytosol. Some parts of high aggregates colocalized with AF488-AD TauO. Scale bar = 10 μm.

**References**

1. Lasagna-Reeves CA, Castillo-Carranza DL, Guerrero-Muoz MJ, Jackson GR, Kayed R. Preparation and characterization of neurotoxic tau oligomers. Biochemistry. 2010;49(47):10039-41.

2. Lo Cascio F, Garcia S, Montalbano M, Puangmalai N, McAllen S, Pace A, et al. Modulating disease-relevant tau oligomeric strains by small molecules. J Biol Chem. 2020;295(44):14807-25.

3. Puangmalai N, Bhatt N, Montalbano M, Sengupta U, Gaikwad S, Ventura F, et al. Internalization mechanisms of brain-derived tau oligomers from patients with Alzheimer's disease, progressive supranuclear palsy and dementia with Lewy bodies. Cell Death Dis. 2020;11(5):314.

4. Kanekiyo T, Xu H, Bu G. ApoE and Abeta in Alzheimer's disease: accidental encounters or partners? Neuron. 2014;81(4):740-54.

5. Lasagna-Reeves CA, Castillo-Carranza DL, Sengupta U, Sarmiento J, Troncoso J, Jackson GR, et al. Identification of oligomers at early stages of tau aggregation in Alzheimer's disease. FASEB J. 2012;26(5):1946-59.

6. Margittai M, Langen R. Template-assisted filament growth by parallel stacking of tau. Proc Natl Acad Sci U S A. 2004;101(28):10278-83.

7. Lasagna-Reeves CA, Glabe CG, Kayed R. Amyloid-beta annular protofibrils evade fibrillar fate in Alzheimer disease brain. J Biol Chem. 2011;286(25):22122-30.
