# Supplementary material for: Lysine 63-linked ubiquitination of tau oligomers contributes to the pathogenesis of Alzheimer’s disease
Source: J Biol Chem. 2022 Feb 22;298(4):101766. doi: 10.1016/j.jbc.2022.101766 (PMC8942844; doi:10.1016/j.jbc.2022.101766)
Supplement: Supplemental Table S1 [file mmc2.doc]

| **Gene names** | **Localization prob** | **Score diff** | **PEP** | **Score** | **Peptide sequence** | **Charge** | **Mass error [ppm]** | **Positions** | **Position** |
| --- | --- | --- | --- | --- | --- | --- | --- | --- | --- |
| MAPT | 1 | 54.09 | 0.009 | 54.09 | AK(gg)TDHGAEIVYK | 3 | 0.822 | 260;354;385 | 385 |
| MAPT | 1 | 46 | 0.011 | 57.17 | TDHGAEIVYK(gg) | 3 | -0.46 | 270;364;395 | 395 |
| MAPT | 1 | 103.8 | 4.02E-11 | 119.5 | IGSLDNITHVPGGGNK(gg) | 2 | -0.59 | 244;338;369 | 369 |
| MAPT | 1 | 98.63 | 0.005 | 106.9 | IGSTENLK(gg) | 2 | 1.867 | 173;267;267 | 267 |
| MAPT | 1 | 92.48 | 0.004 | 94.36 | SK(gg)IGSTENLK | 2 | 0.956 | 165;259;259 | 259 |
| MAPT | 1 | 79.61 | 4E-04 | 79.61 | TPPSSGEPPK(gg)SGDR | 3 | -0.49 | 96;190;190 | 190 |
| MAPT | 1 | 77.54 | 6.51E-07 | 77.54 | ESPLQTPTEDGSEEPGSETSDAK(gg) | 3 | -0.18 | 67;67 | 67 |
| MAPT | 1 | 71.75 | 3.28E-07 | 83.42 | K(gg)DQGGYTMHQDQEGDTDAGLK | 4 | -0.53 | 24;24 | 24 |
| MAPT | 1 | 50.52 | 0.001 | 53.97 | STPTAEDVTAPLVDEGAPGK(gg) | 2 | -0.86 | 87;87 | 87 |
| MAPT | 1 | 143.6 | 2.10E-19 | 157.5 | K(gg)LDLSNVQSK | 2 | -0.44 | 281 | 281 |
| MAPT | 1 | 70.55 | 0.01 | 70.55 | LDLSNVQSK(gg) | 2 | -0.05 | 290 | 290 |
| MAPT | 1 | 40.94 | 3.86E-08 | 91.31 | GAAPPGQK(ac)GQANATR | 3 | -4.43 | 69;163;163 | 163 |
| MAPT | 1 | 64 | 8.61E-05 | 64 | K(ac)DQGGYTMHQDQEGDTDAGLK | 3 | 1.787 | 24;24 | 24 |

**Table S1**. **Identification of ubiquitinated or acetylated Tau peptides**
