# Supplementary material for: Lysine 63-linked ubiquitination of tau oligomers contributes to the pathogenesis of Alzheimer’s disease
Source: J Biol Chem. 2022 Feb 22;298(4):101766. doi: 10.1016/j.jbc.2022.101766 (PMC8942844; doi:10.1016/j.jbc.2022.101766)
Supplement: Supplemental Table S2 [file mmc3.docx]

**Table S2. SRM signature peptides and transitions of Tau, ubiquitin, and ubiquitin linkages**

Q, quadrupole; CE, collision energy.

| **Q1** | **Q3** | **CE** | **protein_name** | **sequence** | **Precursor charge** | **Fragment type** | **Fragment charge** |
| --- | --- | --- | --- | --- | --- | --- | --- |
| 690.3894 | 860.5195 | 27 | Ub-K6-GG | MQIFVK[Ubi]TLTGK | 2 | y7 | 1 |
| 690.3894 | 761.4511 | 27 | Ub-K6-GG | MQIFVK[Ubi]TLTGK | 2 | y6 | 1 |
| 690.3894 | 1007.588 | 27 | Ub-K6-GG | MQIFVK[Ubi]TLTGK | 2 | y8 | 1 |
| 690.3894 | 1120.672 | 26 | Ub-K6-GG | MQIFVK[Ubi]TLTGK | 2 | y9 | 1 |
| 694.3965 | 868.5338 | 27 | Ub-K6-GG | MQIFVK[Ubi]TLTGK[13C6;15N2] | 2 | y7 | 1 |
| 694.3965 | 769.4654 | 27 | Ub-K6-GG | MQIFVK[Ubi]TLTGK[13C6;15N2] | 2 | y6 | 1 |
| 694.3965 | 1015.602 | 27 | Ub-K6-GG | MQIFVK[Ubi]TLTGK[13C6;15N2] | 2 | y8 | 1 |
| 694.3965 | 1128.686 | 26 | Ub-K6-GG | MQIFVK[Ubi]TLTGK[13C6;15N2] | 2 | y9 | 1 |
| 801.4269 | 1002.51 | 35 | ub-K11-GG | TLTGK[Ubi]TITLEVEPSDTIENVK | 3 | y9 | 1 |
| 801.4269 | 1131.552 | 35 | ub-K11-GG | TLTGK[Ubi]TITLEVEPSDTIENVK | 3 | y10 | 1 |
| 801.4269 | 905.4569 | 35 | ub-K11-GG | TLTGK[Ubi]TITLEVEPSDTIENVK | 3 | y8 | 1 |
| 801.4269 | 1230.621 | 35 | ub-K11-GG | TLTGK[Ubi]TITLEVEPSDTIENVK | 3 | y11 | 1 |
| 804.0983 | 1010.524 | 35 | ub-K11-GG | TLTGK[Ubi]TITLEVEPSDTIENVK[13C6;15N2] | 3 | y9 | 1 |
| 804.0983 | 1139.567 | 35 | ub-K11-GG | TLTGK[Ubi]TITLEVEPSDTIENVK[13C6;15N2] | 3 | y10 | 1 |
| 804.0983 | 913.4712 | 35 | ub-K11-GG | TLTGK[Ubi]TITLEVEPSDTIENVK[13C6;15N2] | 3 | y8 | 1 |
| 804.0983 | 1238.635 | 35 | ub-K11-GG | TLTGK[Ubi]TITLEVEPSDTIENVK[13C6;15N2] | 3 | y11 | 1 |
| 701.0389 | 1131.6 | 33 | ub-K27-GG | TITLEVEPSDTIENVK[Ubi]AK | 3 | y9 | 1 |
| 701.0389 | 1016.573 | 34 | ub-K27-GG | TITLEVEPSDTIENVK[Ubi]AK | 3 | y8 | 1 |
| 701.0389 | 915.5253 | 34 | ub-K27-GG | TITLEVEPSDTIENVK[Ubi]AK | 3 | y7 | 1 |
| 701.0389 | 802.4413 | 34 | ub-K27-GG | TITLEVEPSDTIENVK[Ubi]AK | 3 | y6 | 1 |
| 701.0389 | 1315.685 | 31 | ub-K27-GG | TITLEVEPSDTIENVK[Ubi]AK | 3 | y11 | 1 |
| 703.7104 | 1139.614 | 33 | ub-K27-GG | TITLEVEPSDTIENVK[Ubi]AK[13C6;15N2] | 3 | y9 | 1 |
| 703.7104 | 1024.587 | 34 | ub-K27-GG | TITLEVEPSDTIENVK[Ubi]AK[13C6;15N2] | 3 | y8 | 1 |
| 703.7104 | 923.5396 | 34 | ub-K27-GG | TITLEVEPSDTIENVK[Ubi]AK[13C6;15N2] | 3 | y7 | 1 |
| 703.7104 | 810.4556 | 34 | ub-K27-GG | TITLEVEPSDTIENVK[Ubi]AK[13C6;15N2] | 3 | y6 | 1 |
| 703.7104 | 1323.699 | 31 | ub-K27-GG | TITLEVEPSDTIENVK[Ubi]AK[13C6;15N2] | 3 | y11 | 1 |
| 730.8964 | 1016.511 | 28 | Ub-K48-GG | LIFAGK[Ubi]QLEDGR | 2 | y8 | 1 |
| 730.8964 | 959.49 | 28 | Ub-K48-GG | LIFAGK[Ubi]QLEDGR | 2 | y7 | 1 |
| 730.8964 | 1087.549 | 27 | Ub-K48-GG | LIFAGK[Ubi]QLEDGR | 2 | y9 | 1 |
| 730.8964 | 717.3521 | 28 | Ub-K48-GG | LIFAGK[Ubi]QLEDGR | 2 | y6 | 1 |
| 730.8964 | 589.2935 | 28 | Ub-K48-GG | LIFAGK[Ubi]QLEDGR | 2 | y5 | 1 |
| 735.9006 | 1026.52 | 28 | Ub-K48-GG | LIFAGK[Ubi]QLEDGR[13C6;15N4] | 2 | y8 | 1 |
| 735.9006 | 969.4983 | 28 | Ub-K48-GG | LIFAGK[Ubi]QLEDGR[13C6;15N4] | 2 | y7 | 1 |
| 735.9006 | 1097.557 | 27 | Ub-K48-GG | LIFAGK[Ubi]QLEDGR[13C6;15N4] | 2 | y9 | 1 |
| 735.9006 | 727.3603 | 28 | Ub-K48-GG | LIFAGK[Ubi]QLEDGR[13C6;15N4] | 2 | y6 | 1 |
| 735.9006 | 599.3018 | 28 | Ub-K48-GG | LIFAGK[Ubi]QLEDGR[13C6;15N4] | 2 | y5 | 1 |
| 533.7982 | 668.3721 | 21 | Tau | TPSLPTPPTR | 2 | y6 | 1 |
| 533.7982 | 965.5409 | 21 | Tau | TPSLPTPPTR | 2 | y9 | 1 |
| 533.7982 | 781.4561 | 21 | Tau | TPSLPTPPTR | 2 | y7 | 1 |
| 533.7982 | 470.2716 | 21 | Tau | TPSLPTPPTR | 2 | y4 | 1 |
| 538.8023 | 678.3803 | 22 | Tau | TPSLPTPPTR[13C6;15N4] | 2 | y6 | 1 |
| 538.8023 | 975.5492 | 22 | Tau | TPSLPTPPTR[13C6;15N4] | 2 | y9 | 1 |
| 538.8023 | 791.4644 | 22 | Tau | TPSLPTPPTR[13C6;15N4] | 2 | y7 | 1 |
| 538.8023 | 480.2799 | 22 | Tau | TPSLPTPPTR[13C6;15N4] | 2 | y4 | 1 |
| 541.2798 | 665.3611 | 22 | Ubiquitin | TLSDYNIQK | 2 | y5 | 1 |
| 541.2798 | 780.3881 | 22 | Ubiquitin | TLSDYNIQK | 2 | y6 | 1 |
| 541.2798 | 867.4201 | 22 | Ubiquitin | TLSDYNIQK | 2 | y7 | 1 |
| 541.2798 | 980.5042 | 22 | Ubiquitin | TLSDYNIQK | 2 | y8 | 1 |
| 545.2869 | 673.3754 | 22 | Ubiquitin | TLSDYNIQK[13C6;15N2] | 2 | y5 | 1 |
| 545.2869 | 788.4024 | 22 | Ubiquitin | TLSDYNIQK[13C6;15N2] | 2 | y6 | 1 |
| 545.2869 | 875.4344 | 22 | Ubiquitin | TLSDYNIQK[13C6;15N2] | 2 | y7 | 1 |
| 545.2869 | 988.5185 | 22 | Ubiquitin | TLSDYNIQK[13C6;15N2] | 2 | y8 | 1 |
| 894.4672 | 1002.51 | 34 | Ubiquitin | TITLEVEPSDTIENVK | 2 | y9 | 1 |
| 894.4672 | 1131.552 | 32 | Ubiquitin | TITLEVEPSDTIENVK | 2 | y10 | 1 |
| 894.4672 | 818.4249 | 34 | Ubiquitin | TITLEVEPSDTIENVK | 2 | y7 | 1 |
| 894.4672 | 1230.621 | 31 | Ubiquitin | TITLEVEPSDTIENVK | 2 | y11 | 1 |
| 898.4744 | 1010.524 | 34 | Ubiquitin | TITLEVEPSDTIENVK[13C6;15N2] | 2 | y9 | 1 |
| 898.4744 | 1139.567 | 32 | Ubiquitin | TITLEVEPSDTIENVK[13C6;15N2] | 2 | y10 | 1 |
| 898.4744 | 826.4391 | 34 | Ubiquitin | TITLEVEPSDTIENVK[13C6;15N2] | 2 | y7 | 1 |
| 898.4744 | 1238.635 | 31 | Ubiquitin | TITLEVEPSDTIENVK[13C6;15N2] | 2 | y11 | 1 |
