# Supplementary material for: Lysine 63-linked ubiquitination of tau oligomers contributes to the pathogenesis of Alzheimer’s disease
Source: J Biol Chem. 2022 Feb 22;298(4):101766. doi: 10.1016/j.jbc.2022.101766 (PMC8942844; doi:10.1016/j.jbc.2022.101766)
Supplement: Supplemental Table S3 [file mmc4.docx]

**Table S3. Summary of human cases examined in this study**

| **Pathology/ Brain #** | **Age** | **Gender** | **Post-mortem interval (h)** | **Brain area** | **Braak stage** |
| --- | --- | --- | --- | --- | --- |
| Control 1 | 76 | Female | 2 | Frontal cortex | I |
| Control 2 | 86 | Female | 1.75 | Frontal cortex | I |
| Control 3 | 79 | Male | 1.75 | Frontal cortex | II |
| AD 1 | 77 | Male | 4.5 | Frontal cortex | VI |
| AD 2 | 83 | Male | 3.5 | Frontal cortex | VI |
| AD 3 | 82 | Female | 3.1 | Frontal cortex | VI |
