# Supplementary material for: Lysine 63-linked ubiquitination of tau oligomers contributes to the pathogenesis of Alzheimer’s disease
Source: J Biol Chem. 2022 Feb 22;298(4):101766. doi: 10.1016/j.jbc.2022.101766 (PMC8942844; doi:10.1016/j.jbc.2022.101766)

Raw file	Scan	Method	Score	m/z	Gene names
AD-Tau1	4110	FTMS; HCD	54.09	482.58	MAPT

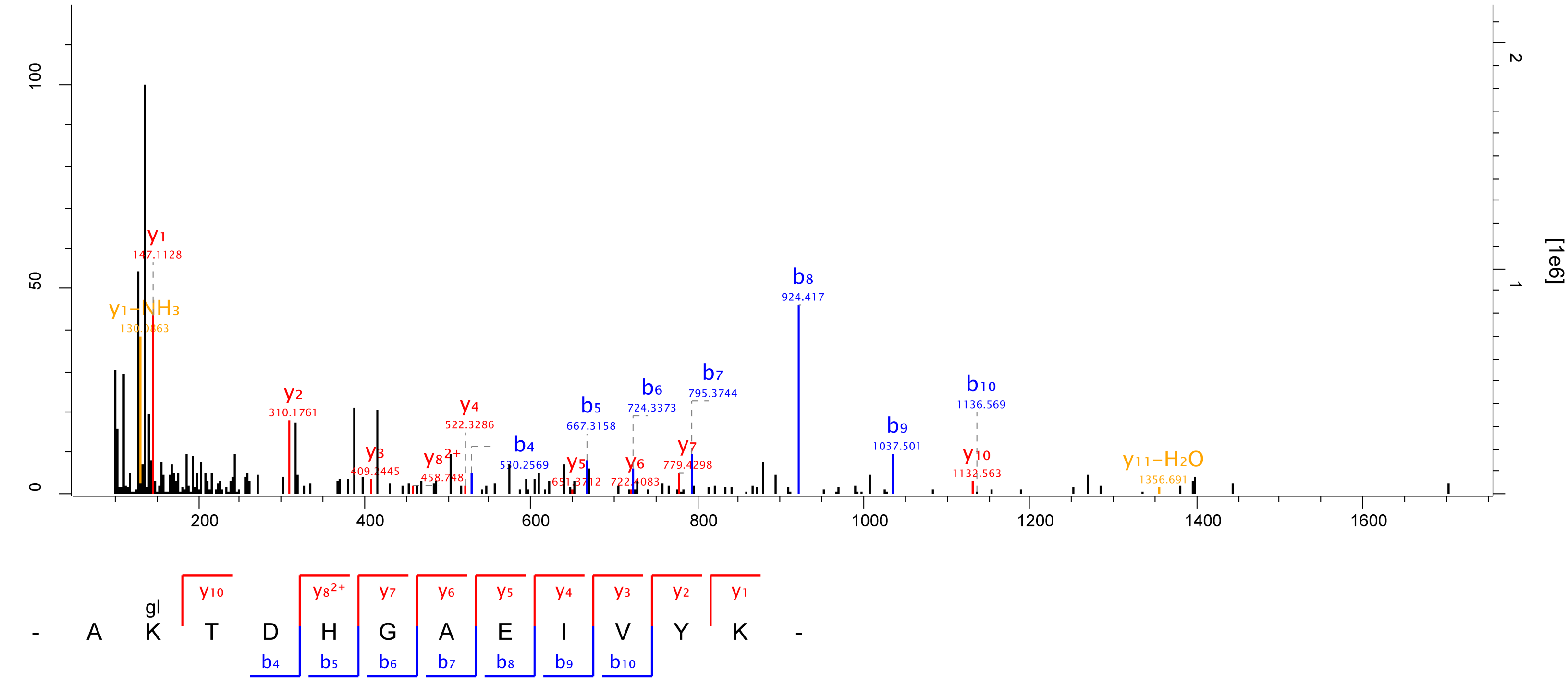

Raw file	Scan	Method	Score	m/z	Gene names
AD-Tau1	5608	FTMS; HCD	46	416.21	MAPT

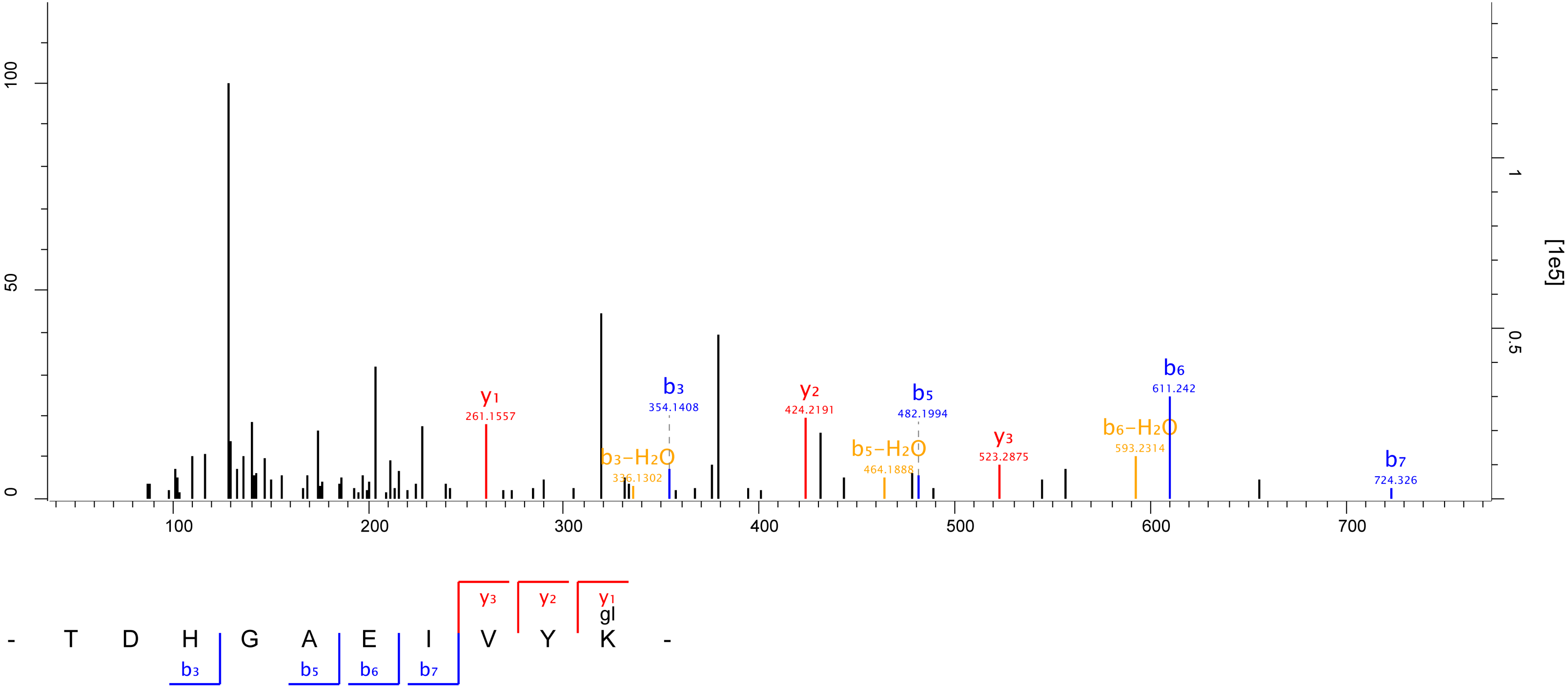

Raw file	Scan	Method	Score	m/z	Gene names
AD-Tau2	5775	FTMS; HCD	103.76	846.93	MAPT

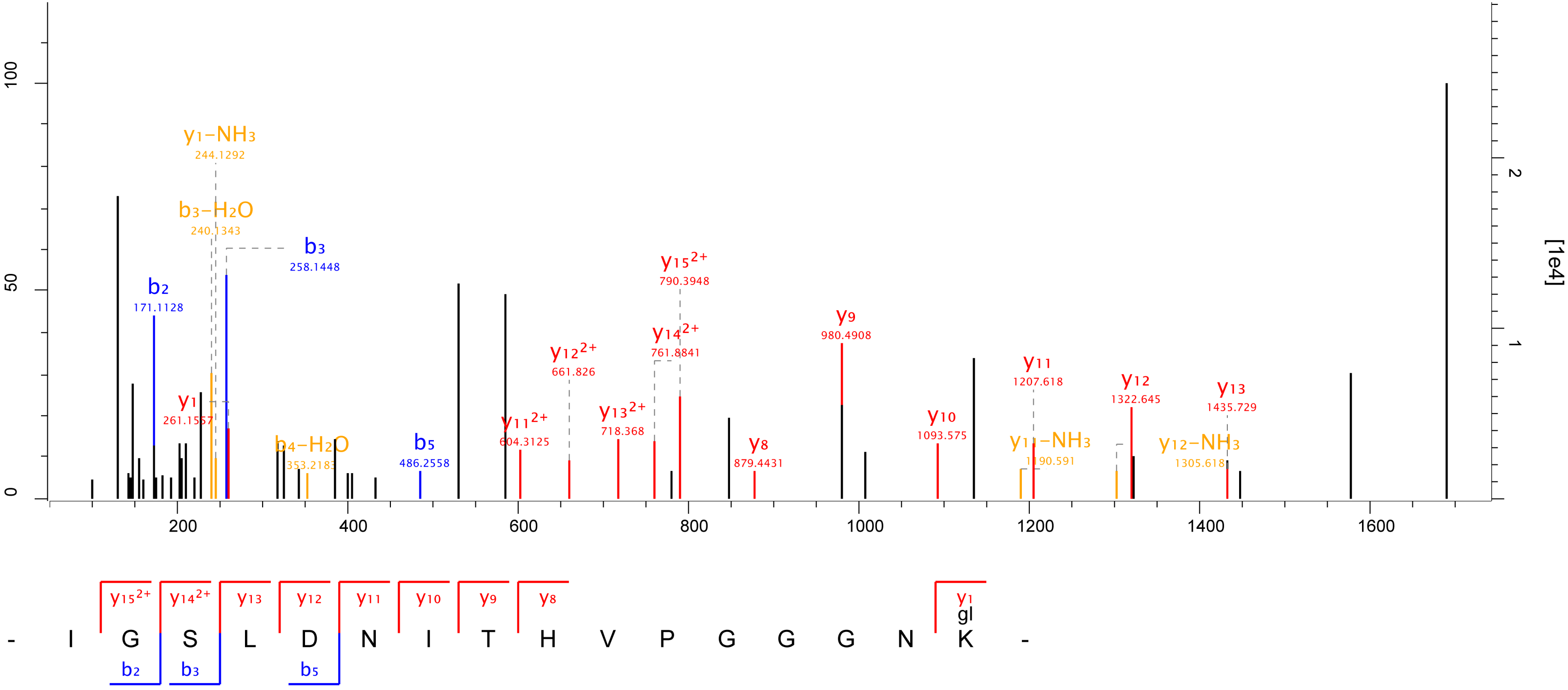

Raw file	Scan	Method	Score	m/z	Gene names
AD-Tau2	3092	FTMS; HCD	98.63	488.26	MAPT

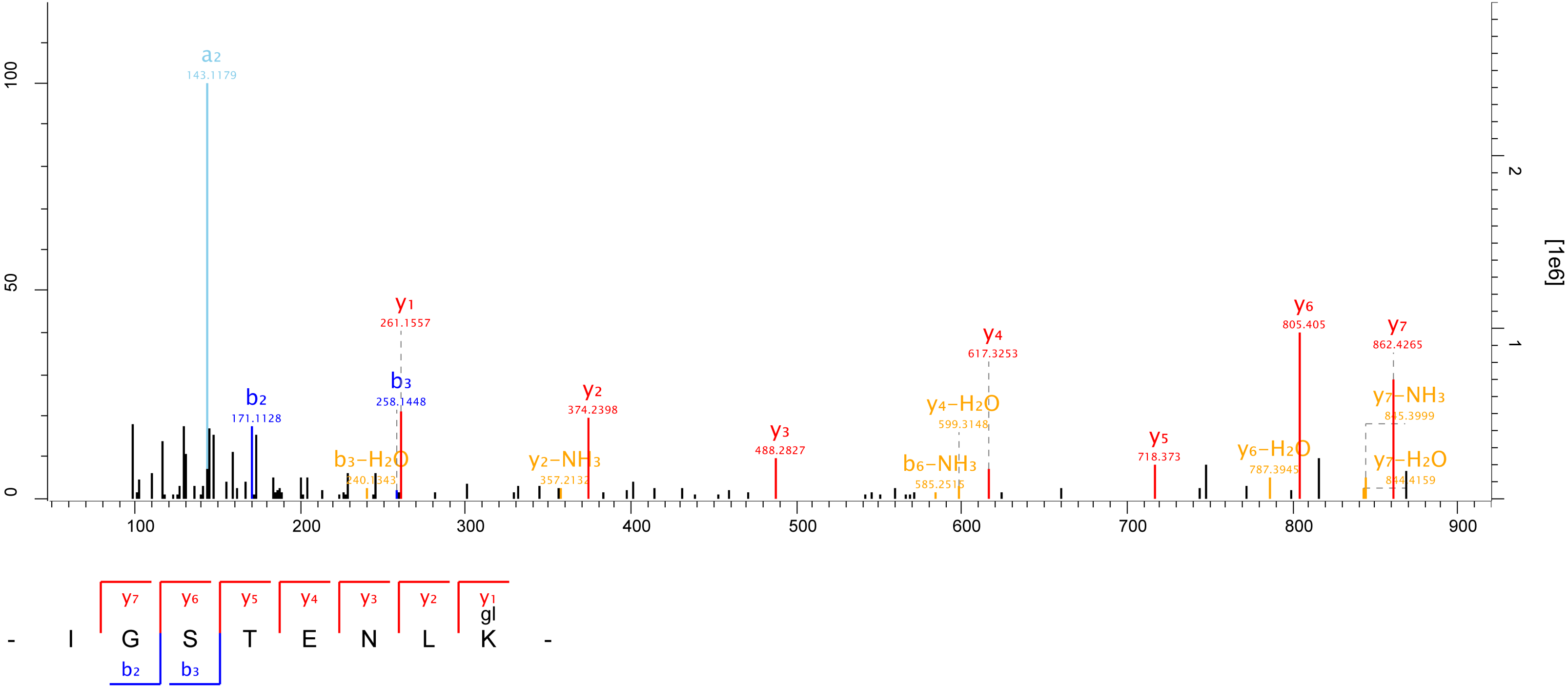

Raw file	Scan	Method	Score	m/z	Gene names
AD-Tau1	3879	FTMS; HCD	94.36	595.82	MAPT

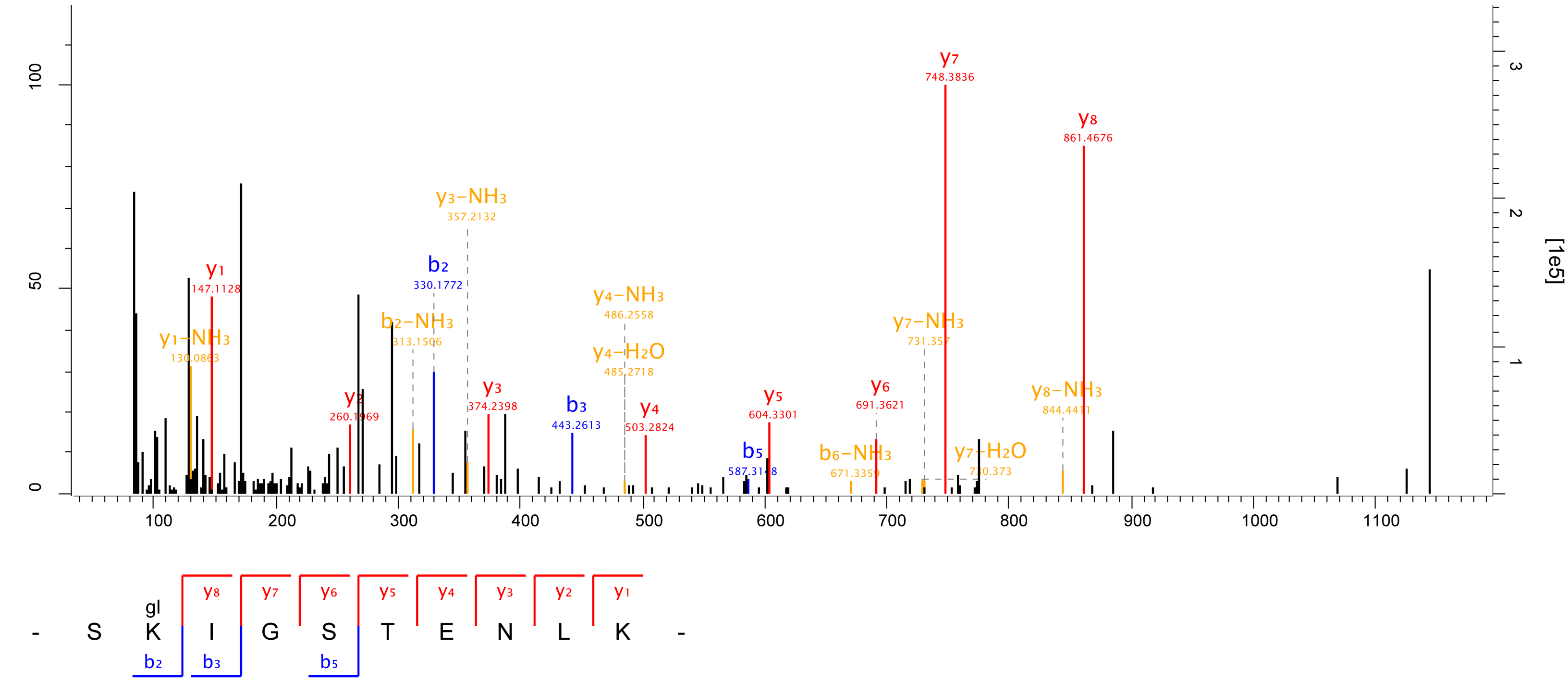

Raw file	Scan	Method	Score	m/z	Gene names
AD-Tau1	2941	FTMS; HCD	79.61	509.24	MAPT

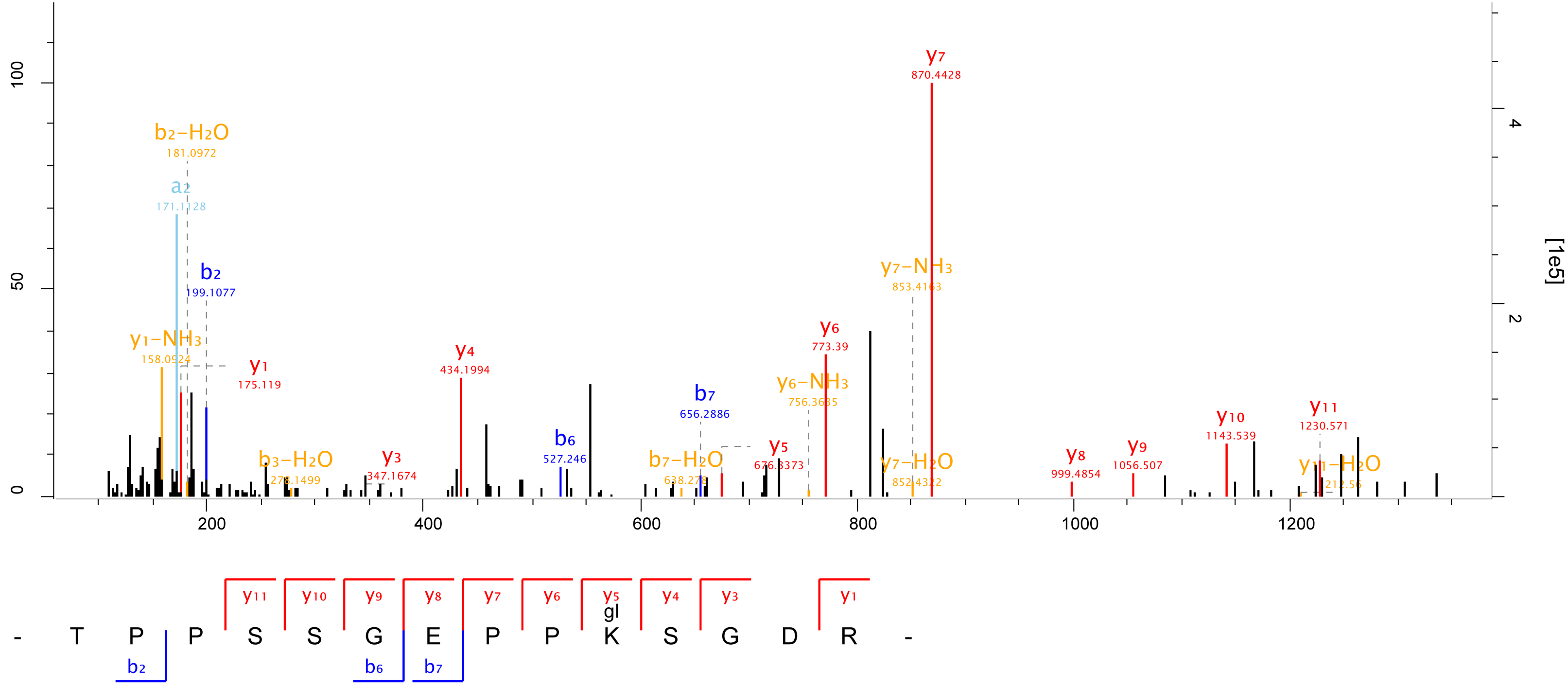

Raw file	Scan	Method	Score	m/z	Gene names
AD-Tau1	8256	FTMS; HCD	77.54	836.03	MAPT

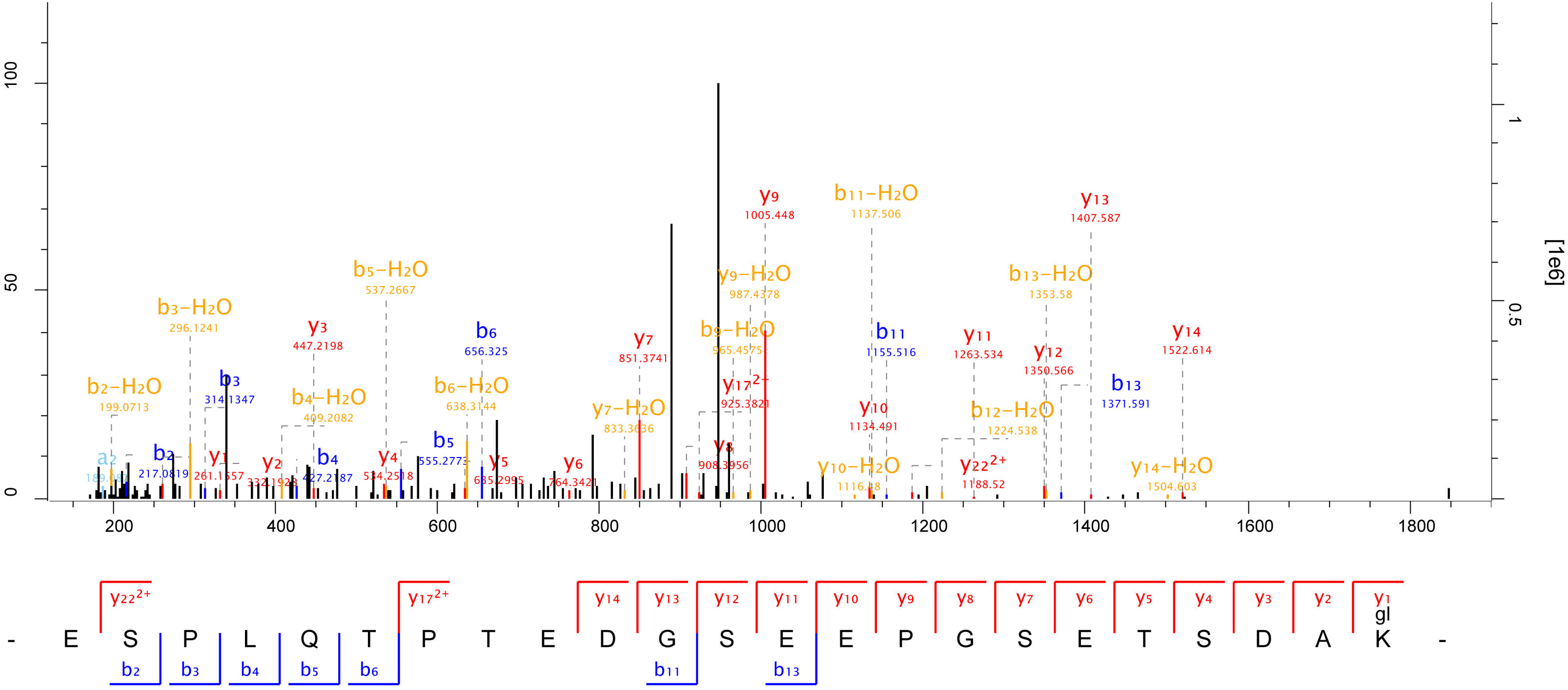

Raw file	Scan	Method	Score	m/z	Gene names
AD-Tau1	4749	FTMS; HCD	83.42	607.01	MAPT

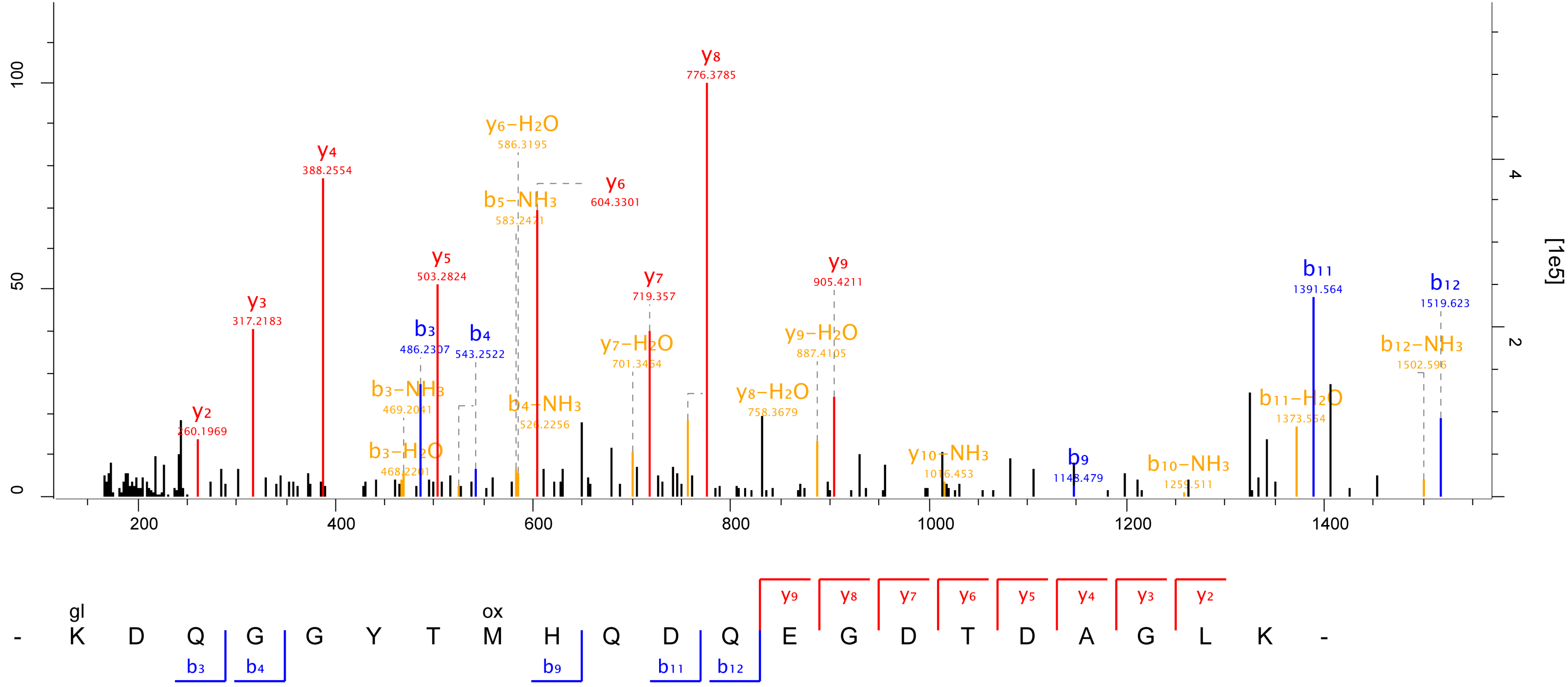

Raw file	Scan	Method	Score	m/z	Gene names
AD-Tau2	7112	FTMS; HCD	50.52	1035	MAPT

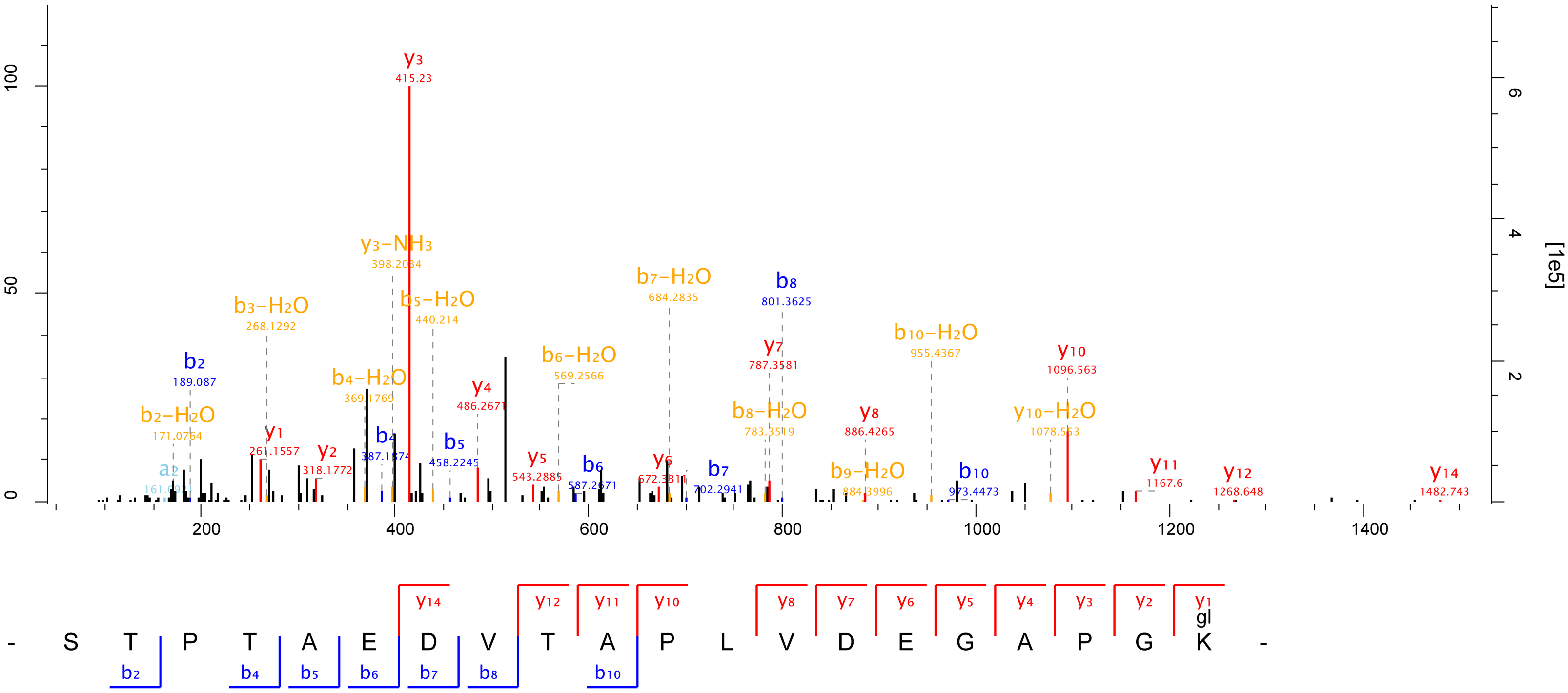

Raw file	Scan	Method	Score	m/z	Gene names
AD-Tau1	7803	FTMS; HCD	157.54	623.34	MAPT

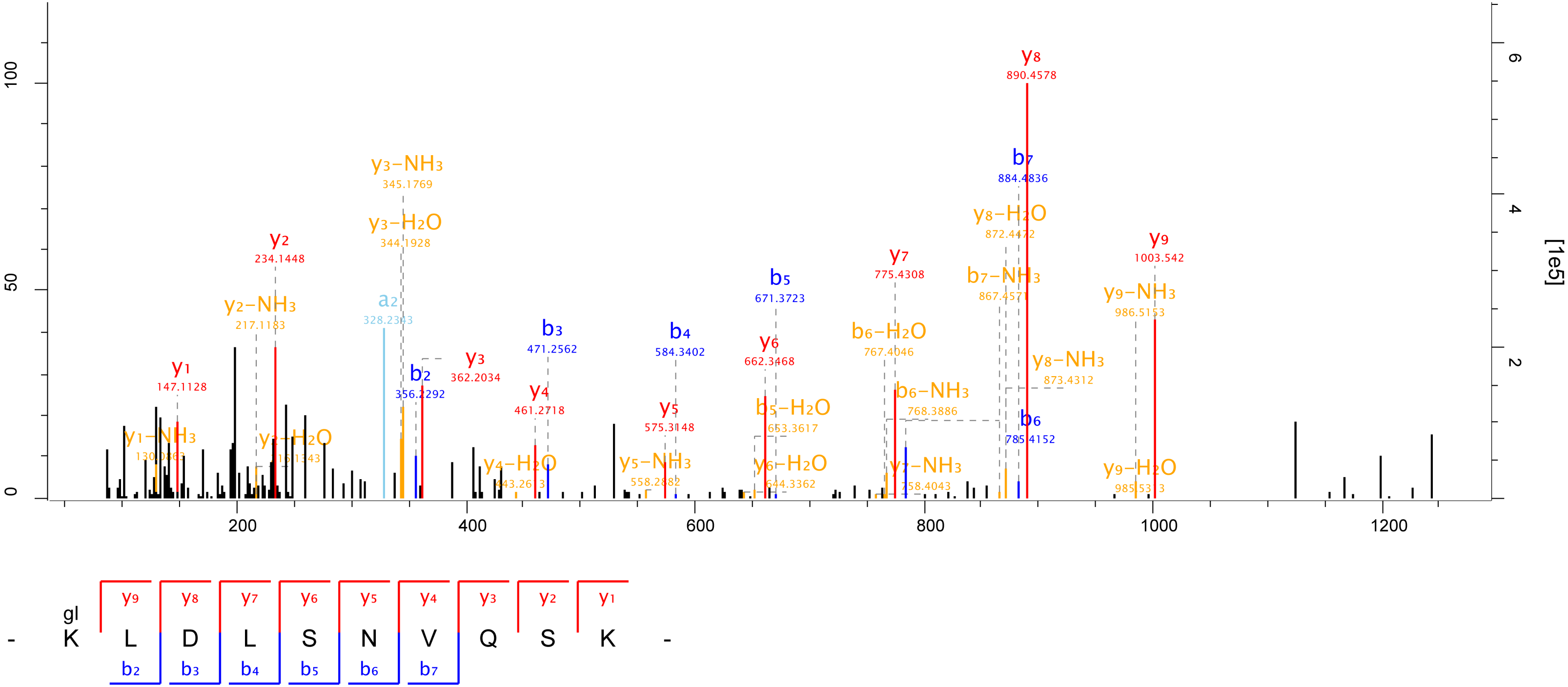

Raw file	Scan	Method	Score	m/z	Gene names
AD-Tau1	7496	FTMS; HCD	70.55	559.3	MAPT

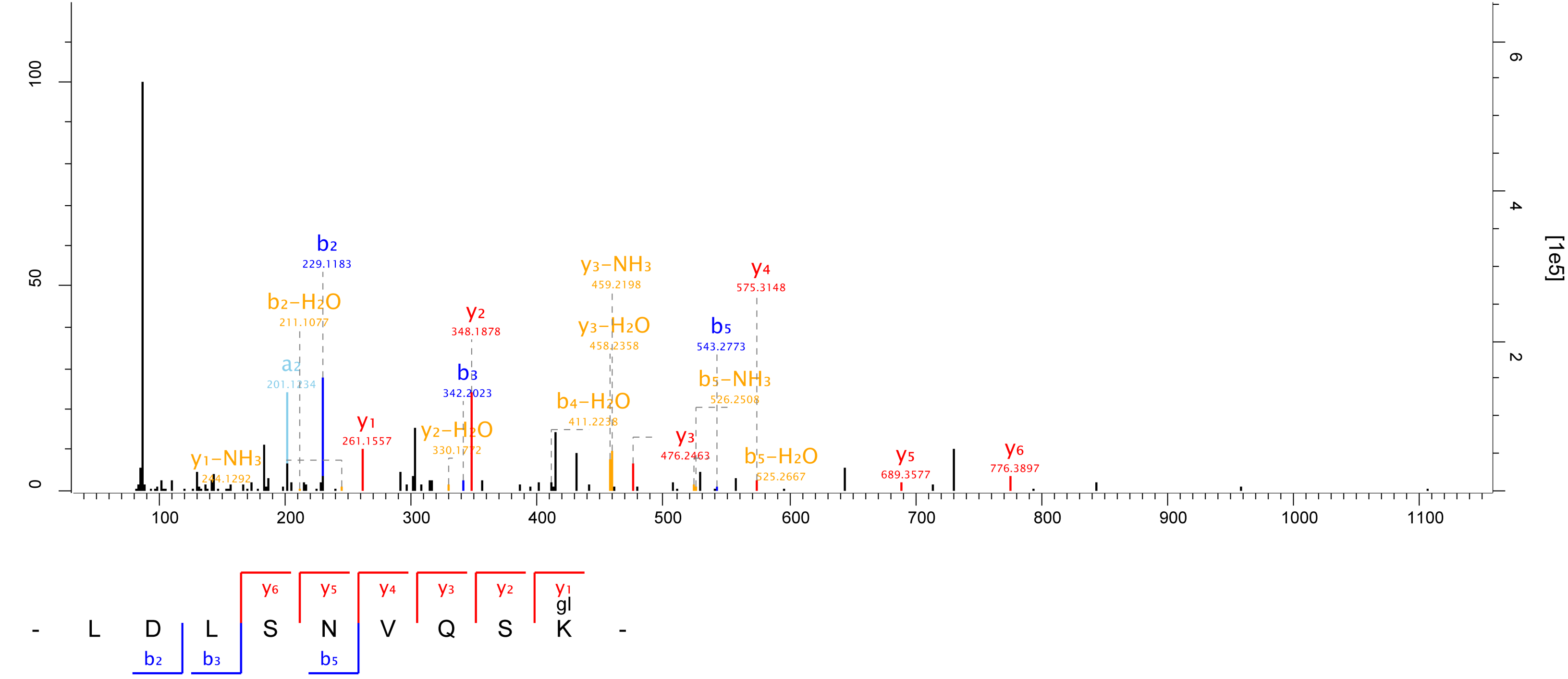

Raw file	Scan	Method	Score	m/z	Gene names
AD-Tau1	3114	FTMS; HCD	91.31	489.25	MAPT

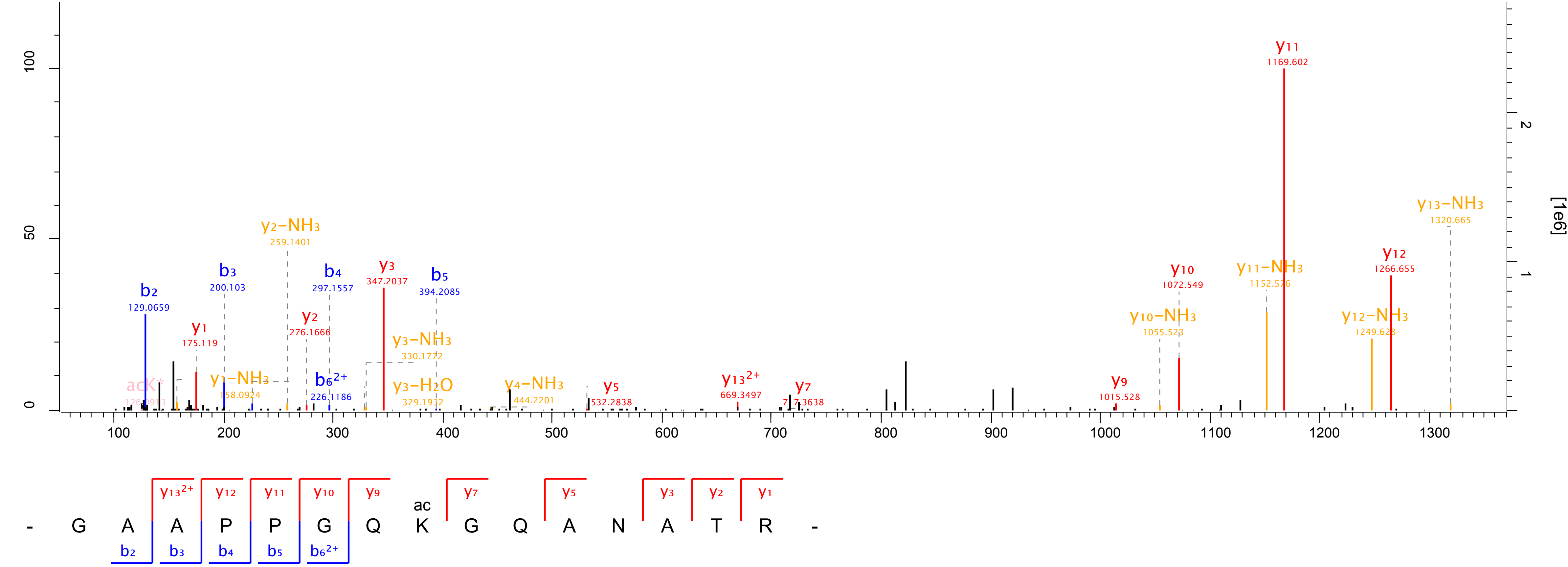

Raw file	Scan	Method	Score	m/z	Gene names
AD-Tau2	3738	FTMS; HCD	64	784.67	MAPT

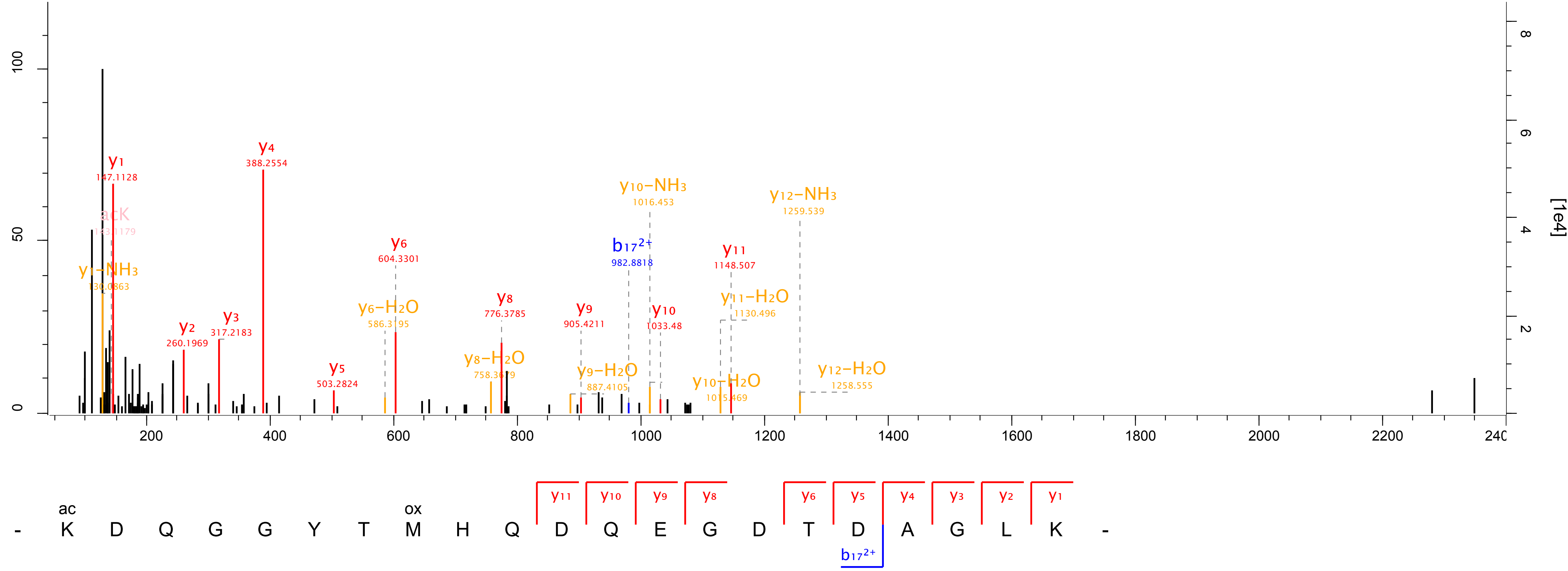

Supplement: Supplemental Figure S7 [file mmc5.pdf]
